# An FBXW7-ZEB2 axis links EMT and tumour microenvironment to promote colorectal cancer stem cells and chemoresistance

**DOI:** 10.1038/s41389-019-0125-3

**Published:** 2019-02-19

**Authors:** Ningning Li, Roya Babaei-Jadidi, Federica Lorenzi, Bradley Spencer-Dene, Philip Clarke, Enric Domingo, Eugene Tulchinsky, Robert G. J. Vries, David Kerr, Yihang Pan, Yulong He, David O. Bates, Ian Tomlinson, Hans Clevers, Abdolrahman S. Nateri

**Affiliations:** 1 Cancer Genetics and Stem Cell Group, Cancer Biology, Division of Cancer and Stem Cells, School of Medicine, University of Nottingham, Nottingham, NG7 2UH UK; 2 The Seventh Affiliated Hospital of Sun Yat-sen University, 518107 Shenzhen, China; 3 The Institute of Cancer Research, Surrey, SM2 5NG UK; 4 Advanced Cell Diagnostics, Henry Wellcome Building of Genomic Medicine, Oxford, OX3 7BN UK; 5 Cancer Biology Unit, Division of Cancer and Stem Cells, School of Medicine, University of Nottingham, Nottingham, NG7 2UH UK; 6 Wellcome Trust Centre for Human Genetics, Henry Wellcome Building of Genomic Medicine, Oxford, OX3 7BN UK; 7 Department of Cancer Studies, University of Leicester, Leicester, UK; 8 Moscow Institute of Physics and Technology, Dolgoprudny, Moscow region, Russia; 9 Hubrecht Institute for Developmental Biology and Stem Cell Research, Utrecht and University Medical Centre Utrecht, Uppsalalaan 8, 3584CT Utrecht, Netherlands; 10 John Radcliffe Hospital, Nuffield Division of Clinical Laboratory Sciences, Oxford, OX3 9DU UK

## Abstract

Colorectal cancer (CRC) patients develop recurrence after chemotherapy owing to the survival of stem cell-like cells referred to as cancer stem-like cells (CSCs). The origin of CSCs is linked to the epithelial–mesenchymal transition (EMT) process. Currently, it remains poorly understood how EMT programmes enable CSCs residing in the tumour microenvironment to escape the effects of chemotherapy. This study identifies a key molecular pathway that is responsible for the formation of drug-resistant CSC populations. Using a modified yeast-2-hybrid system and 2D gel-based proteomics methods, we show that the E3-ubiquitin ligase FBXW7 directly binds and degrades the EMT-inducing transcription factor ZEB2 in a phosphorylation-dependent manner. Loss of FBXW7 induces an EMT that can be effectively reversed by knockdown of ZEB2. The FBXW7-ZEB2 axis regulates such important cancer cell features, as stemness/dedifferentiation, chemoresistance and cell migration in vitro, ex vivo and in animal models of metastasis. High expression of ZEB2 in cancer tissues defines the reduced ZEB2 expression in the cancer-associated stroma in patients and in murine intestinal organoids, demonstrating a tumour-stromal crosstalk that modulates a niche and EMT activation. Our study thus uncovers a new molecular mechanism, by which the CRC cells display differences in resistance to chemotherapy and metastatic potential.

## Introduction

About 40–50% of patients with stage II and stage III colorectal cancer (CRC) exhibit resistance to therapy and develop recurrent cancer over the course of treatment^1^. CRC cells respond to transcriptional and epigenetic changes and undergo epithelial–mesenchymal transition (EMT). In cancer, the EMT is associated with the cell capacity to self-renew (termed cancer stem-like cells (CSCs)), generating different lineages of cells (tumour heterogeneity) and resistance to therapies and metastasis^2^. Environmental factors control the CSC properties. However, few studies exist to provide a clear mechanistic understanding of how the development of migrating CRC-CSCs (CR-CSCs) and drug resistance are related to the tumour microenvironment.

E3-ubiquitin ligases (E3s) form a talented class of regulators. The specificity of proteolysis is determined by the association of a specific E3-receptor subunit with the substrate. FBXW7 (also called hCDC4, Fbw7) functions as a receptor subunit for the Skp1/Cullin/F-box (SCF)-E3 (SCF^FBXW7^) and targets several proteins with critical roles in the hallmarks of cancer^3,4^. Thus, elucidating the FBXW7 mechanism(s) of action can add valuable information for identifying therapeutic targets and strategies to block CRC growth and metastasis. We and others have previously engineered mice in which the *Fbxw7* gene is conditionally knocked out in the intestine (*fbxw*7^ΔG^), resulting in accelerated tumorigenesis in *Apc*^Min^-mutant mice as early as 2–5 weeks after birth^5,6^. These studies highlight a possibility that FBXW7 was associated with the intestinal/colon stem cells (ISCs). However, because of the early lethality of *Apc*^Min^*fbxw*7^ΔG^ mice, little is known about the role of FBXW7 in CR-CSC growth and metastasis.

ISC-associated signalling pathways are often affected in CRC-SCs^7,8^. As in ISCs, the self-renewal and survival signals dominate over the differentiation signals in CRC-SCs. Hence, we hypothesised that FBXW7 may exert its function by degrading proteins expressed in ISCs and that the loss of FBXW7 may endow them with the functional hallmarks of CR-CSCs. To explore this further, we identified Fbxw7-associated proteins (FAPs) that were expressed in crypt/ISC-isolation followed by 2D-MALDI-MS, which were also phosphorylation-dependent targets of FBXW7 using the yeast-based, cytoplasmic two-hybrid Ras-Recruitment-System (RRS) assays^9^. Here, we focus on a master regulator of EMT, the Zinc-finger E-box-binding homeobox-2(ZEB2) transcription factor protein (also known as SIP1 and Zfhx1b)^10,11^ as a new GSK-3β phosphorylation-dependent target of FBXW7.

ZEB2 has previously been implicated in EMT, cell-cycle progression, apoptosis and senescence^10,12–16^. ZEB2 was overexpressed in bladder, ovarian, stomach, pancreatic and squamous cell carcinoma, in the intestinal subtype of stomach cancers, and at the invasive front of CRC where EMT is most prominent^17–20^. ZEB2 also mediates cell-fate decision in neuronal, T cells and hematopoietic stem cells^21–23^. In this study, we addressed how the FBXW7-ZEB2 axis mediates an interplay between EMT, cancer-associated fibroblasts (CAFs) and CR-CSCs and regulates CRC metastasis and chemoresistance.

## Results

### ZEB2 degradation via its physical interaction with FBXW7

**Fig. 1:**
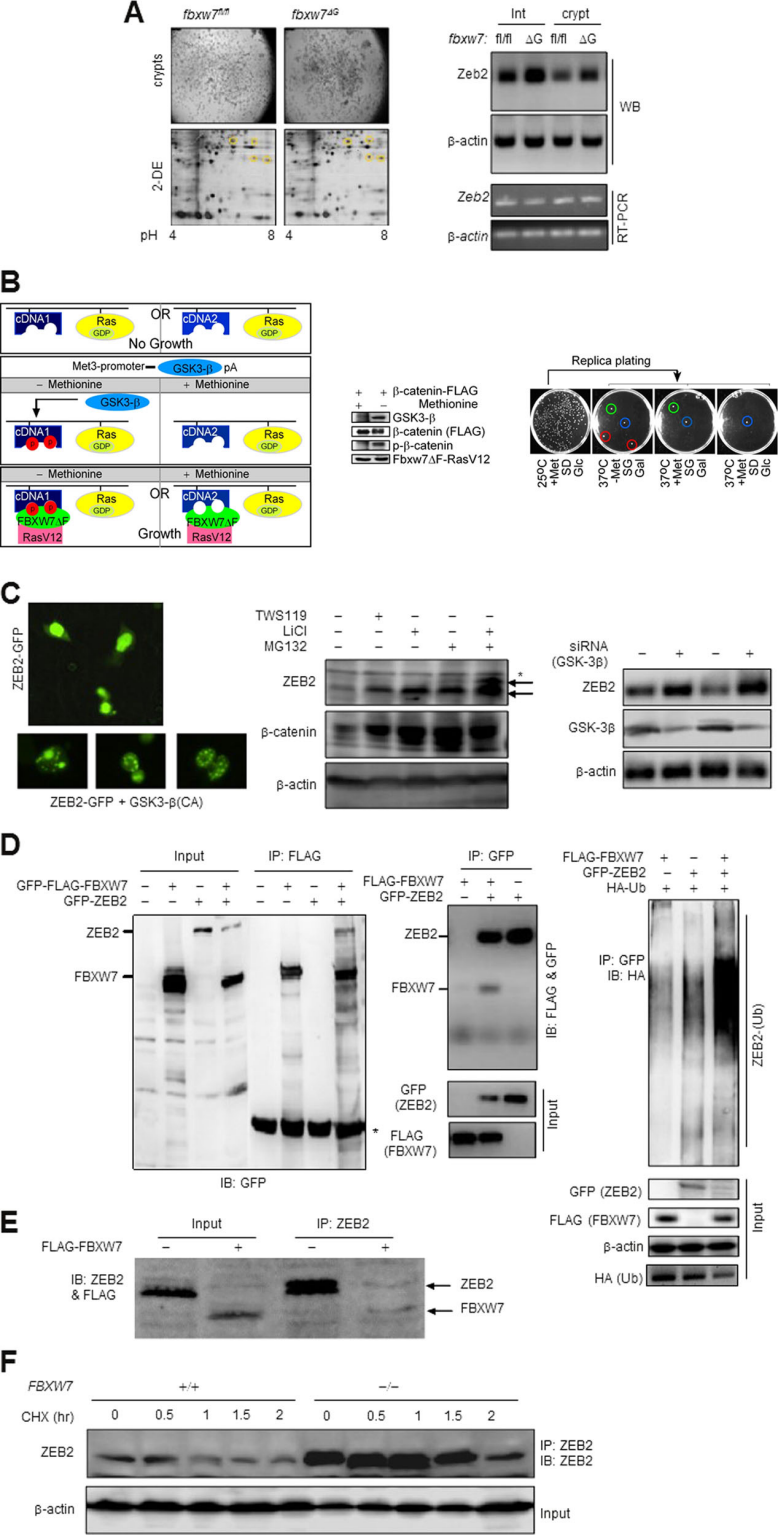
SCF^FBXW7^ interacts and targets ZEB2 for degradation in a GSK-3β phosphorylation-dependent manner. **a** Left, 2DE and MALDI-MS-based identification of novel Fbxw7-associated proteins using crypts (upper panel) isolated from 3-week *fbxw*7^fl/fl^ and *fbxw*7^ΔG^ mice. Yellow circles in the lower panel denote potential Fbxw7-associated proteins. **a** Right, WB analysis (upper panels), and RT-PCR analysis (lower panels) of *fbxw*7^fl/fl^ vs. *fbxw*7^ΔG^ derived crypts and intestinal proteins and mRNA expression for ZEB2 and β-actin control. Experiments were performed on at least three independent occasions. **b** Left, schematic representation of the modified yeast two-hybrid reverse Ras Recruitment Screening (rRRS) system identifying proteins interacting with Fbxw7 in a GSK-3β phosphorylation-dependent manner. GSK-3β under the control of the methionine-regulated *MET*3 promoter induces phosphorylation of encoded myristoylated proteins through a cDNA library plus positive control expressing FLAG-β-catenin (B—Middle) which only rescued the growth of cdc25–2 mutant yeast by Fbxw7-associated protein(s), if they interact with RasV12-FBXW7ΔF (i.e. human FBXW7α isoform mutant lacking F-box domain; therefore, interaction with Skp1 is lost and degradation of SCF^Fbxw7^ substrates will not occur in yeast) used as a bait at the restrictive temperature 37 °C, in a methionine-dependent manner. In the FBXW7ΔF mutant, both the N-terminal F-box and Dim-domains are deleted to avoid any interactions with SKP1 and other FBXW7 isoform-associated proteins. Thus, cdc25–2 mutant yeasts can grow only at 37 °C, when a phosphorylation-dependent interaction between a protein target and RasV12-FBXW7ΔF takes place. The FBXW7ΔF(bait)-dependent growth of these clones was further analysed on galactose-containing medium at 37 °C (B—Right). Red circles show the GSK-3β-phosphorylation-dependent interactor, including the Zeb2-clone, green circles show the phosphorylation/non-phosphorylation-dependent interactor and blue circles show the revertant clones (B—Right). **c** Left, subcellular localisation of GFP-fused human ZEB2 in the absence (top; nuclear) and presence (bottom; nuclear spots indicative of protein degradation) of GSK-3β in HCT116 CRC cells. (**c—**Middle and **c—**Right) WB analysis of total ZEB2 protein level following the inhibition of GSK-3β (e.g. WS119 or LiCl treatment, and siRNA against GSK-3β) and of UPS pathways (MG132) in SW620 CRC cells. **d** Direct binding and ubiquitin-dependent degradation of ZEB2 by FBXW7. Co-immunoprecipitation (IP) of ZEB2 upon pull-down of FBXW7 in HEK-293T cells (Left); co-IP of FBXW7 upon pull-down of ZEB2 using the TNT-coupled reticulocyte lysate (Middle), and ubiquitination assays with HA-tagged ubiquitin- (HA-Ub) expressing construct in HEK-293T cells (Right). The asterisk indicates a nonspecific band(s). **e** Co-IP of endogenous ZEB2 upon pull-down of FBXW7 in HCT116 cells with *FBXW*7 deletion. **f** ZEB2 pulse-chase stability assays with 15 µg/ml cycloheximide (CHX) in HCT116 cells with or without *FBXW*7 deletion

To investigate the Fbxw7 function in ISCs, we isolated the control “floxed” *fbxw7* (*fbxw*7^fl/fl^) and mutant *fbxw*7^ΔG^ intestinal crypts^24^. Proteins, either absent in control *fbxw*7^fl/fl^ or upregulated in homozygous *fbxw*7^ΔG^ and heterozygous *fbxw*7^ΔG/+^, were initially identified by 2D/MALDI-TOF mass spectrometry (Fig. 1a, left, and Table S1, significance threshold *p* < 0.05). Because the SCF^FBXW7^ targets multiple substrates, it may indirectly affect the abundance and phosphorylation status of different proteins on the 2D gel. Thus, we established a yeast two-hybrid reverse Ras-recruitment system (RRS)^9^ (Fig. 1b). Proteins detected in both RRS and 2D-MALDI-MS assays, and with no previously known links with FBXW7, including ZEB2 (Table S1), were further investigated.

Upon the RRS analyses, 12 out of 219 plasmids rescued in the absence of methionine, encoded various fragments of the ZEB2 C-terminus (between 515 and 1100aa). Also, increased expression of the ZEB2 protein (>3 times) but not the corresponding mRNA was detected in *fbxw*7^∆G^ versus *fbxw*7^fl/fl^ crypts (Fig. 1a, right). Hence, we performed further experiments to test if GSK-3β/FBXW7 negatively regulates ZEB2 in vitro. When GFP-ZEB2 was overexpressed in different cell lines, we found different patterns of GFP-ZEB2 expression in response to GSK-3β activation (Fig. 1c, left). Reciprocally, treatment with either TWS119 or LiCl (potent GSK-3β inhibitors), MG132 (an inhibitor of the 26 S proteasome) (Fig. 1c, middle) or GSK-3β knockdown (Fig. 1c, right), upregulated ZEB2 expression. Furthermore, since there is no anti-phospho-site-specific antibody to detect phosphorylated ZEB2, we examined GSK-kinase activity-mediated ZEB2 phosphorylation by using the endogenous ZEB2 immunoprecipitates when LiCl and BIO inhibit GSK-3 activity in FBXW7-deficient cells. These data suggest that GSK-inhibitor treatment results in significant inhibition of S/T-phosphorylated ZEB2 (Figures S1C and S2A).

We next examined whether ZEB2 is a direct target of SCF^FBXW7^. As FBXW7 isoform-specific antibodies are unavailable that work for endogenous immunoprecipitation (IP) and western blotting (WB) assays, and FBXW7α is the most abundant isoform expressed in the intestine, we used this isoform for follow-up studies. Co-IP experiments revealed that exogenous and endogenous FBXW7 and ZEB2 proteins bind with each other in cells (Fig. 1d, e left, and S2B and S2C). IP of TNT-coupled reticulocyte lysate of FLAG-Fbxw7 also showed a direct interaction with ZEB2 (Fig. 1d, middle). We assessed ZEB2 ubiquitination in HEK293 and CRC cells and a typical high-molecular-mass smear of exogenous and endogenous ubiquitinated ZEB2 precipitated in the presence of FBXW7 (Fig. 1d, right and S1A). Also, the cycloheximide pulse-chase experiment revealed a total absence of endogenous and exogenous ZEB2 protein degradation in the absence of FBXW7 (Fig. 1f and S1B). These data demonstrate that the ZEB2 phosphorylation could be crucial for SCF^FBXW7^-mediated ZEB2 destabilisation.

### Mechanism of ZEB2 degradation by FBXW7

**Fig. 2:**
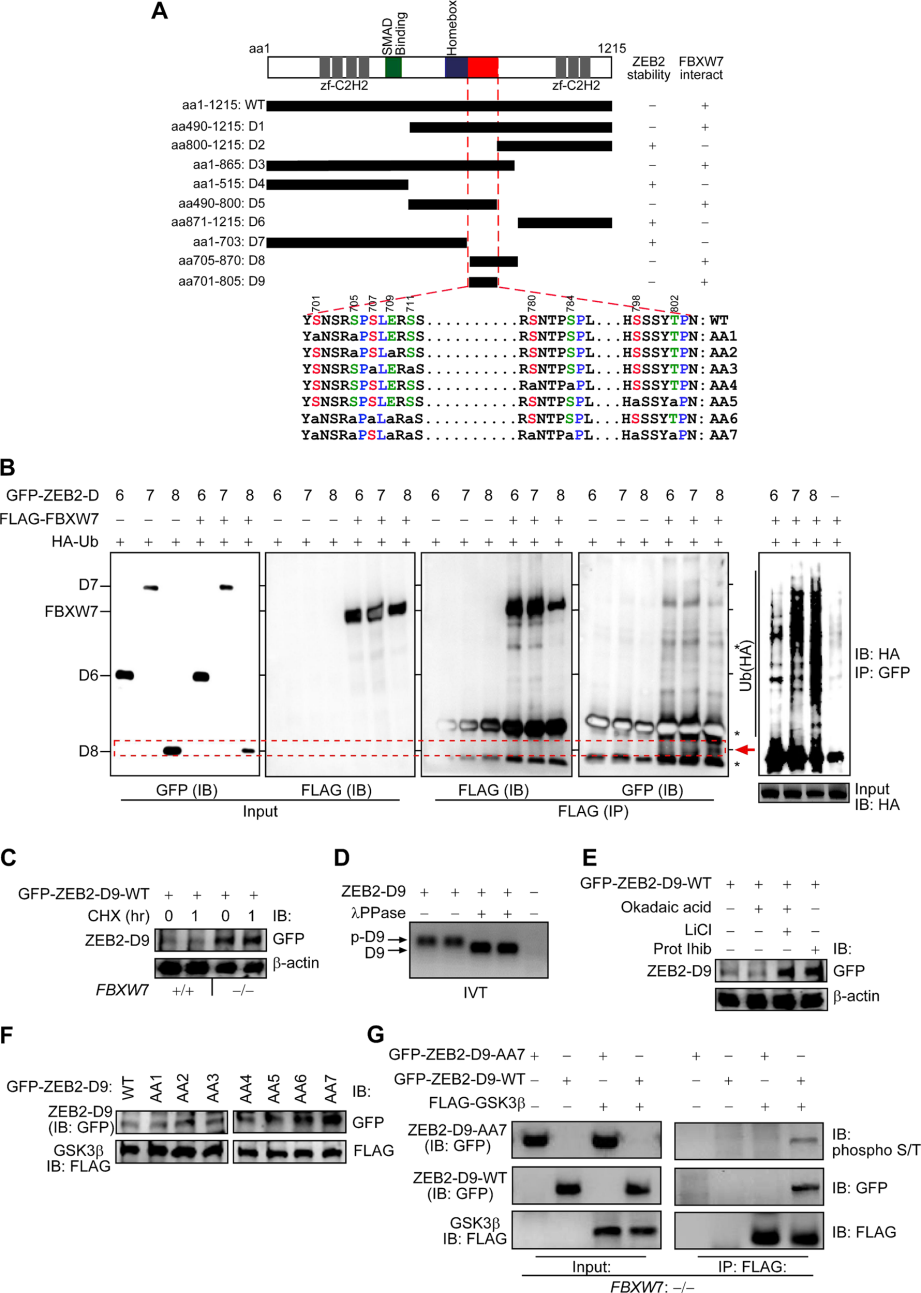
GSK-3-mediated phosphorylation-dependent degradation of ZEB2 by FBXW7α. **a** Schematic mapping and identifying FBXW7 phosphodegrons on ZEB2 protein. Constructs of D1–D9 represent the structure of the GFP-fused ZEB2 deletions. Serine and threonine residues within the potential GSK-3β phosphorylation sites (i.e. degron sequences) are shown in red and green, and proline residues are in blue in wild-type ZEB2-D9; whilst the small letter “a” indicates S/T residues replaced by alanine in the mutant ZEB2-D9 (AA1 to AA7, where AA1 + AA2 + AA3 = AA6, and AA4 + AA5 + AA6 = AA7). **b** ZEB2-D8 directly binds to FBXW7 for ubiquitin and GSK-3β-mediated degradation. HEK-293T cells transfected with the indicated constructs (D6–D8) together with FLAG-GSK-3β plasmid and HA-tagged ubiquitin (HA-Ub) followed by IP and IB. The red arrowhead (fourth panel) denotes the co-IP of the ZEB2-D8 mutant in the FBXW7 precipitates. Co-IP and IB experiments were performed in triplicate. **c** FBXW7 controls the degradation of ZEB2-D9, the shorter version of ZEB2-D8. HCT116 cells ±FBXW7 were transfected with the ZEB2-D9 construct, treated with cycloheximide (CHX) for 1 h and whole-cell lysates were subjected to IB. **d** Phosphorylation of ZEB2-D9 may be a prerequisite for its degradation. Lambda protein phosphatase (λPPase) treatment leads to faster motility due to the release of phosphate groups from phosphodegrons. **e** ZEB2-D9 protein stability depends on phosphorylation and proteasome. HEK-293T cells were transfected with the ZEB2-D9 construct, treated with Okadaic acid (inhibitor of PP1 and PP2A phosphatases; lane2), LiCl (GSK-3β inhibitor; lane3) or MG132 proteasome inhibitor I (Prot Inhib; lane4) for 8 h and whole-cell lysates were subjected to IB. **f** Phosphodegrons within ZEB2-D9 are collectively essential to its stability. HEK-293T cells were transfected with wild-type (WT) ZEB2-D9 and D9-phosphorylation-defective mutants (AA1–AA7 constructs) together with FLAG-GSK-3β plasmid and whole-cell lysates subjected to IB. **g** HCT116^FBXW7(−/−)^ cells were transfected with GFP-ZEB2-D9 wild-type (WT) and mutant (AA7) and the activated FLAG-GSK3β. FLAG-GSK3β was immunoprecipitated with anti-FLAG and then detected with the phospho-S/T antibody. GFP-ZEB2-D9 phosphorylation status was examined by immunoblot analysis after immunoprecipitation using an anti-phospho-(Ser/Thr) antibody that efficiently detected phospho wild-type GFP-ZEB2-D9-WT

The protein sequence of ZEB2 (NCBI: CCDS2186.1) contains four potential conserved CPDs [the FBXW7/(Cdc4)-phosphodegron sequence (T/S)P/L-X-X-(S/T/D/E)]^4^ among vertebrates and five putative GSK-3β phosphorylation sites (Figures S1D and S1E). To interrogate the ZEB2-specific domains in FBXW7-mediated degradation, we initially constructed eight GFP-ZEB2 deletion mutants (D1–D8) (Fig. 2a) and measured their expression levels in the presence or absence of FBXW7. In WB analysis, the ZEB2-D2, ZEB2-D4, ZEB2-D6 and ZEB2-D7 mutants were not, or only slightly affected by FBXW7, as compared with the ZEB2-D1, ZEB2-D3, ZEB2-D5 and ZEB2-D8 proteins (Figures S1F, S1G and 2B, 1st blot). Intriguingly, amino-acid sequence comparison revealed that the D1, D3, and D8 mutants contained adjacent CPDs and putative GSK-3β phosphorylation sites within the area between S705 and T802, which increased protein instability (Fig. 2a, b, 1st blot). In comparison with D1, D3, and D8 mutants, the D5 mutant lacks the potential phosphorylation of threonine 802 that may correlate with a slightly enhanced ZEB2-D5 stability affected by FBXW7 (Figs. S1F and 2a, b, 1st blot). Also, co-IP showed that ZEB2-D8 interacted with FBXW7 (Fig. 2b, 4th blot, red arrowhead) and heavily ubiquitinated (Fig. 2b, 5th blot and S3A). These data indicate that CPDs and phospho-motifs within the homeobox-C-terminal region and adjacent to the CtBP-binding motif^16^ are required for ZEB2 degradation. However, we encountered difficulties when trying to generate the full-length ZEB2 mutants of each phosphorylation site, due to lack of proper restriction enzyme(s). To overcome this, we used the combined overlap extension PCR and eventually obtained a full-length ZEB2 lacking the aa705–870 (ZEB2-ΔD8) (Figure S3B). Next, to determine if the -ΔD8 mutant stabilises the protein, we performed CHX chases, and this further confirmed that aa705–870 residues contribute to destabilisation of the ZEB2 protein (Figure S3C).

Next, we constructed a ZEB2-D9 expression, containing the CPDs and phospho-motifs (700−804aa) (Fig. 2a). Consistently, the stability of the ectopic expression of the ZEB2-D9 protein was restored in HCT116^FBXW7(−/−)^ cells (Fig. 2c and S1H). Moreover, TNT-coupled reticulocyte lysate of ZEB2-D9 showed that phosphorylation occurred at the ZEB2 C-terminus domain, displaying altered electrophoretic mobility, while treatment with λ-phosphatase resulted in a non-phosphorylated, faster-migrating form (Fig. 2d). To investigate the significance of each phosphorylation site, we constructed phospho-incompetent GFP-ZEB2-D9 mutants by converting each Serine and/or Threonine to an Alanine (Fig. 2a). The ZEB2-D9 with S/T → A mutations, particularly at the residues S779, S797 (AA4 mutant), S784 and T802 (AA5 mutant), effectively stabilised the proteins both in the presence of GSK-3β or proteasome inhibitors (Fig. 2e, f and S1H). Quantifying western blot data normalised to β-actin, we demonstrated statistically significant differences in phospho-mutants versus wild-type D9 constructs (Figure S1H). Furthermore, we showed that GSK-3β kinase directly phosphorylates ZEB2 (Fig. 2g). Thus, the S/T-rich domain is a core regulatory region responsible not only for the ZEB2/FBXW7 interaction but also for FBXW7-mediated GSK-3β-dependent ZEB2 degradation.

### ZEB2 promotes EMT and cell invasion in colorectal cancer cells

**Fig. 3:**
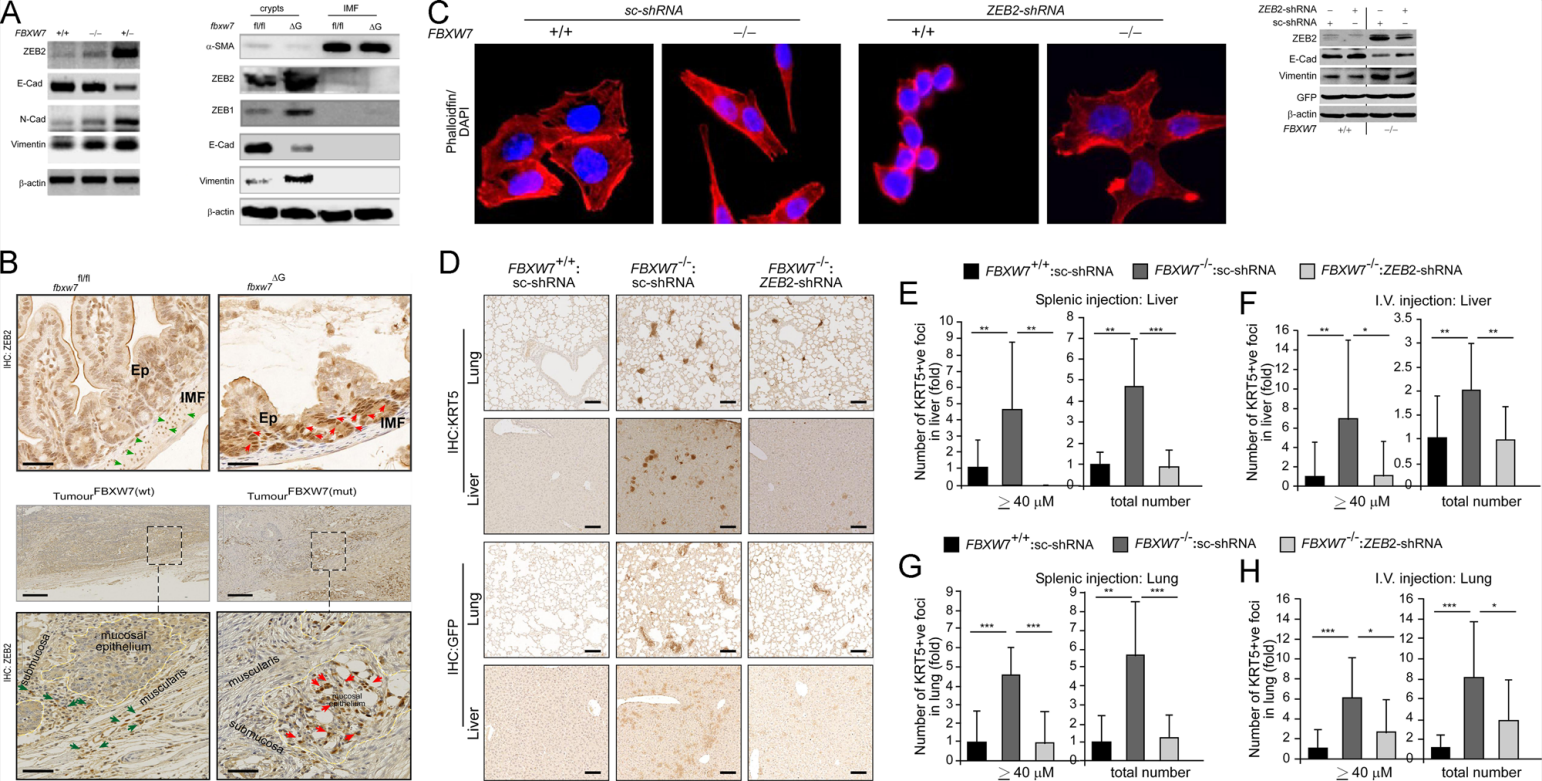
Aberrant ZEB2 expression induces EMT, migration and invasion of CRC cells in vitro and in vivo. **a** WB analysis of DLD1 cells ± FBXW7 (left) and murine *fbxw*7^fl/fl^ vs. *fbxw*7^ΔG^ derived crypts and IMF proteins (right) using α-SMA, ZEB2, Vimentin, N-cadherin, E-cadherin antibodies, and β-actin loading control. **b** Top, ZEB2 IHC on the intestine from 3-week *fbxw*7^fl/fl^ and *fbxw*7^ΔG^ mice. Dashed lines indicate the boundary of the IMF and Ep. Red arrowheads show Ep and green arrowheads show IMF with different Zeb2 protein levels in *fbxw*7^fl/fl^ vs. *fbxw*7^ΔG^. **b** Bottom, IHC for ZEB2 in samples of CRC patients with (*n* = 10) and without (*n* = 11) *FBXW7* mutations. A boxed line indicates a magnified tissue area. Red arrowheads show Ep and green arrowheads show stromal cells with different ZEB2 protein levels. Scale bars, 50 μm. **c** Left, HCT116^FBXW7(−/−)^ and HCT116^FBXW7(+/+)^ cells with ZEB2 knockdown (ZEB2-shRNA) and scrambled vector (sc-shRNA) controls, stained with rhodamine–phalloidin marking F-actin filaments. Scale bars, 100 µm. **c** Right, WB analysis of HCT116 cells ± FBXW7, expressing the sc-shRNA controls and ZEB2-shRNA using ZEB2, Vimentin and E-cadherin antibodies. **d** Representative images of xenograft metastatic models containing disseminated sc-shRNA:FBXW7(+/+), sc-shRNA:FBXW7(−/−) and ZEB2-shRNA:FBXW7(−/−) HCT116 cells in the murine liver and lung. Tissues were stained with antibodies against human keratin5 (KRT5) (top panels) or against the cell tag GFP (bottom panels). Scale bars, 50 µm. **e**–**h** Total number of foci of disseminated cells or foci with size ≥40 µm of sc-shRNA:FBXW7(+/+), sc-shRNA:FBXW7(−/−) and ZEB2-shRNA:FBXW7(−/−) HCT116 cells in the liver (**e**, **f**) and lung (**g**, **h**) were manually counted in five views of KRT5 stained sections/mouse and per each cell line. Absolute number was normalised to control sc-shRNA:FBXW7(+/+) cell line. Bars represent mean ± SD, *n* = 5; *P < 0.05, **P < 0.01, ***P < 0.001, using Student’s *t* test

We initially analysed the level of several proteins known as FBXW7 targets. WB assays showed that the level of c-Myc and P100 was unchanged, but HIF-1α, MCL-1 and KLF5 increased in murine *fbxw*7^∆G^ mutant crypts, while the level of KLF5 and c-Myc increased in CRC cells (Figure S4A). Also, previous reports showed no significant accumulation of phosphorylated c-Myc, cyclin-E and/or β-catenin at 5–6 weeks of age in *fbxw*7^ΔG^ mice^5,6,25^. Given these findings, we sought to study the molecular links between FBXW7 loss, ZEB2 and the consequent changes in the intestine and CRC cells using several models. Interestingly, both WB and IF assays verified that homozygous or heterozygous *FBXW7* knockout in CRC cells augmented ZEB2 protein levels (e.g. Fig. 3a, left, S4B and S4C), and in murine *fbxw*7^∆G^ crypts versus *fbxw7*^fl/fl^ controls (Fig. 3a, right). In contrast, *ZEB2* mRNA and miR200 expression levels were unchanged (Figure S5, D–F), indicating that FBXW7 did not affect the signalling pathways regulating *ZEB2* transcription or mRNA degradation. However, the immunohistochemistry (IHC) analysis demonstrated substantial expression of the ZEB2 protein in epithelial cells but not in the intestinal myofibroblasts (IMF) of *fbxw7*^∆G^ mice. In contrast, a strong ZEB2 immunopositivity was detected in IMF cells, but not in the cells of the intestinal epithelium in *fbxw7*^fl/fl^ controls (Fig. 3b, top, green and red arrowheads). Consistently, in patients’ samples harbouring *FBXW7* mutations, ZEB2 expression was higher in epithelial cells than in stroma, while in samples with wild-type FBXW7, the expression pattern was opposite (Fig. 3b, bottom, and S5A, green and red arrowheads). These findings were irrespective of the genetic background of the tumours (MSI, type of *FBXW7* mutation and grade and stage of a tumour). Although due to the low number of samples, no statistically significant correlation between ZEB2 protein and patient’s metastasis-free or overall survival was assessed. The study of patients’ samples further confirmed the differences in the ZEB2 expression between the epithelium and stroma detected in mouse intestinal tissues.

To investigate whether the ZEB2-expression pattern has an effect on the functioning of the immune system, we isolated CD4 + T cells (i.e. essential mediators of immune homeostasis and inflammation) from the intestinal lamina propria (LP) of *fbxw*7^ΔG^ and *fbxw*7^fl/fl^ mice as previously described^26^. The number of CD4 + T cells in different individual mice varied, but statistical analysis revealed no significant difference between mutant and control groups (n = 7/group) (Figure S5B). These results suggest that the intestinal *FBXW7* mutation resulting in aberrant expression of ZEB2 may alter tumorigenicity via the EMT and potential changes in the interactions between epithelial cells and IMF with no effect on the intestinal immunity.

HCT116^FBXW7(−/−)^ and DLD1^FBXW7(−/−)^ cells failed to form confluent monolayers with intercellular conjunctions, and exhibited elongated, spindle shapes (Fig. 3c, left). Consistent with a recent report^27^, an increased Vimentin, N-cadherin, ZEB1, Snail1 and reduced E-cadherin expression levels were found both in FBXW7^(−/−)^ CRC cell lines and *fbxw*7^∆G^ crypts, suggesting that FBXW7 depletion induces EMT (e.g. Fig. 3a, c right, S5C, S5D and S6 A-D). *ZEB2* knockdown led to a restoration of rounded morphology (Fig. 3c, left and S6B), enhanced E-cadherin and reduced Vimentin expression (e.g. Fig. 3c, right, S6A and C–F). As ZEB1 and Snail1 also upregulated in FBXW7-depleted protein lysates, the ZEB2 knockdown had partial effects in regulating E-cadherin and Vimentin (Fig. 3c, right and S6C–D). Also, FBXW7 depletion in CRC cells resulted in faster in vitro wound closure and migration, while *ZEB2* knockdown attenuated cell migration (Figure S7, A–C).

Furthermore, the ZEB2-ΔD8 overexpression had no effects on E-cadherin and Vimentin protein levels in HCT116 cells (Figure S3D). These data further confirm the effect of FBXW7/ZEB2 interaction on EMT. Next, we investigated FBXW7/ZEB2 roles on the migration/invasion and metastatic potential of CRC cells in vivo. The scrambled (sc)-GFP:HCT116^FBXW7(+/+)^, sc-GFP:HCT116^FBXW7(–/–)^ and ZEB2-shRNA^K/D^:HCT116^FBXW7(−/−)^ cells expressing *luciferase*, were injected directly into the spleen and/or the tail vein of 15 immunodeficient mice and tracked by bioluminescent imaging (Figure S7D). Mice were regularly imaged every 14 days to monitor the formation and growth of the tumours. Mice were terminated after 2 months when cysts/tumours became detectable (Figure S7E). Following termination, livers and lungs were isolated (Figure S7F) and processed for IHC analyses. IHC with monoclonal anti-human KRT5 or anti-GFP antibodies^28^, marked only human cells in tumours (Figure S7G). The anti-KRT5 staining showed more intensive and confirmed the presence of the human CRC cells in mouse organs (Fig. 3d, top vs. bottom). A higher number and larger size of metastatic foci were detected in the lungs and livers (the most common site of metastasis in CRC patients), in mice injected with HCT116^FBXW7(−/−)^ cells, while the metastatic ability of ZEB2-shRNA^K/D^:HCT116^FBXW7(−/−)^ cells was relatively low (Fig. 3e–h). These findings from both in vitro and in vivo experiments suggest that *ZEB2* knockdown significantly reduced tumour cell motility and the incidence of liver and lung metastasis.

### FBXW7/ZEB2-induced EMT inhibits the apoptotic response to chemotherapy which can be abolished by fibroblasts

**Fig. 4:**
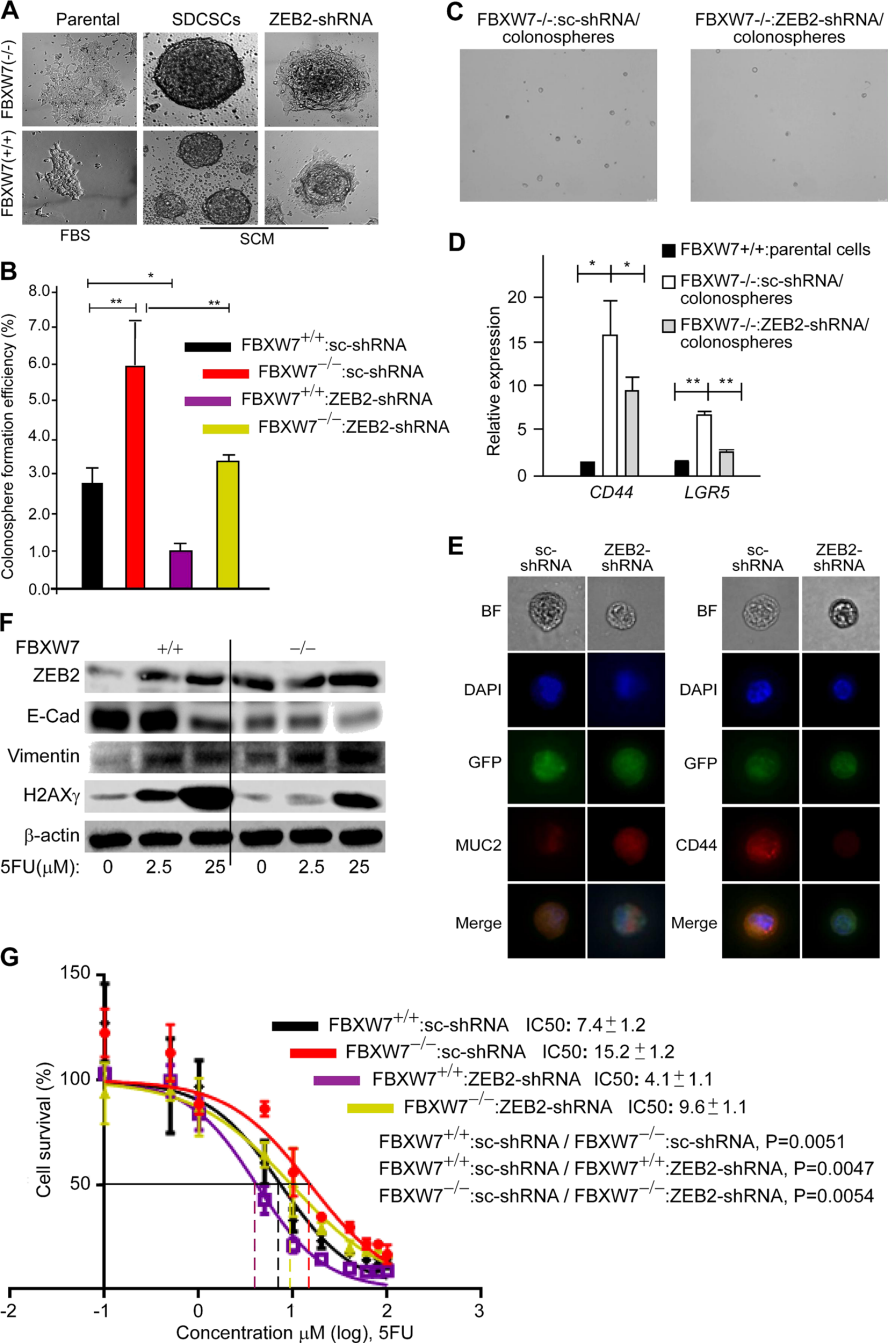
ZEB2/EMT signalling increases chemoresistance and stemness driven by the *FBXW*7 mutation in human CRC cells. **a** Representative images of sphere-derived cancer stem-like cells (SDCSCs) and sphere-derived adherent cells by sc-shRNA and ZEB2-shRNA expressing cell lines. **b** Quantification of the colonosphere-forming ability of the above cell lines. FBS foetal bovine serum, SCM serum-free stem cell medium. **c** Representative images of colonospheres derived from sc-shRNA:FBXW7(−/−) and ZEB2-shRNA:FBXW7(−/−) cells. **d** qRT-PCR analysis of colorectal cancer and intestinal stem cell markers, *CD*44 and *LGR*5, in ZEB2-shRNA:FBXW7(−/−) colonospheres, compared with sc-shRNA:FBXW7(−/−) controls (*n* = 50 *P < 0.05, **P < 0.01). **e** Immunofluorescence analysis of Mucin2 (MUC2, differentiation marker) and CD44 in ZEB2-shRNA:FBXW7(−/−) colonospheres, compared with sc-shRNA:FBXW7(−/−) controls (*n* = 15). **f** EMT markers, ZEB2, E-cadherin and Vimentin, and a DNA double-strand break marker, Gamma-H2AX (γH2AX) are measured at a low (2.5 μM) and a high (25 μM) dose of (5-FU) in synchronised/serum- starved HCT116^FBXW7(+/+)^ and HCT116^FBXW7(−/−)^ cells by WB analysis. **g** Survival of synchronised/serum-starved sc-shRNA:FBXW7(+/+), ZEB2-shRNA:FBXW7(+/+), sc-shRNA:FBXW7(−/−) and ZEB2-shRNA:FBXW7(−/−) HCT116 cell lines is assessed after treatment with 10 increasing doses of 5-FU by SRB colorimetric assay, performed in triplicate for each cell line on three independent occasions. IC50 values, calculated by using GraphPad Prism software 7.02, represent the mean of three different experiments ± SEM. *P* values (~0.005) between *sc*-shRNA and ZEB2-shRNA expressing cell lines with the same and different FBXW7 status using the AIC approach in Prism by comparing two datasets (curves) at a time

To elucidate the consequences of FBXW7/ZEB2-induced EMT on CR-CSC biology, as CR-CSC markers are very heterogeneous, we studied the in vitro colonosphere model of CR-CSCs^29,30^. Consistent with the previous report, FBXW7 loss increased^27^, and that *ZEB*2 knockdown decreased the colonosphere size and efficiency compared with FBXW7^(−/−)^ cells (Fig. 4a–c). Moreover, *ZEB2* knockdown reduced the expression of stemness genes (*Lgr*5, *CD*44) but increased the expression of the differentiation marker MUC2 in colonospheres (Fig. 4d, e). As stemness associated with chemoresistance^24,27,31,32^, we examined the effect of *ZEB2* knockdown on FBXW7-deficiency-induced chemoresistance. WBs and cytotoxicity analyses of ZEB2-shRNA^K/D^:HCT116^FBXW7(−/−)^ cells further confirmed that induction of ZEB2/EMT through the loss of SCF^FBXW7^-E3-ligase activity induced resistance to 5-fluorouracil (5-FU) and Oxaliplatin (OX) chemotherapeutics in CRC cells (Fig. 4f, g and S6G). Furthermore, the colony-forming efficiency of 5-FU ZEB2-shRNA^K/D^ chemoresistant cells (generated by exposing the cells to increasing concentrations of 5-FU for 2–3 months) was compromised, compared with the parental cell lines after exposure to 5-FU (Figure S8A). These results demonstrated that ZEB2-induced EMT mediates the maintenance of drug resistance and CR-CSC properties.

That fibroblasts and tumour cells appeared to be able to interact and crosstalk reciprocally (Fig. 3b), we investigated the effect of CAFs and normal human fibroblasts (NFs) on ZEB2-mediated drug resistance of CRC cells. HCT116^FBXW7(−/−)^ cells were cocultured with either NFs (express high-level ZEB2) or CAFs while treated with different doses of 5-FU. When cultured alone, wild-type and *FBXW7*-mutant HCT116 cells were about 6- and 14 times, respectively, more resistant to 5-FU than the fibroblasts (Figure S8B, black and red logline vs. green logline). Of note, HCT116^FBXW7(−/−)^ sensitivity to 5-FU significantly increased when cells were cocultured with NFs but not with CAFs (Figure S8B, blue logline). Thus, NFs may sensitise drug-resistant epithelial-derived CRC cells to chemotherapy, whereas CAFs may promote chemoresistance.

### ZEB2-induced EMT and stromal markers promote tumorigenesis in *fbxw*7-mutated organoids

**Fig. 5:**
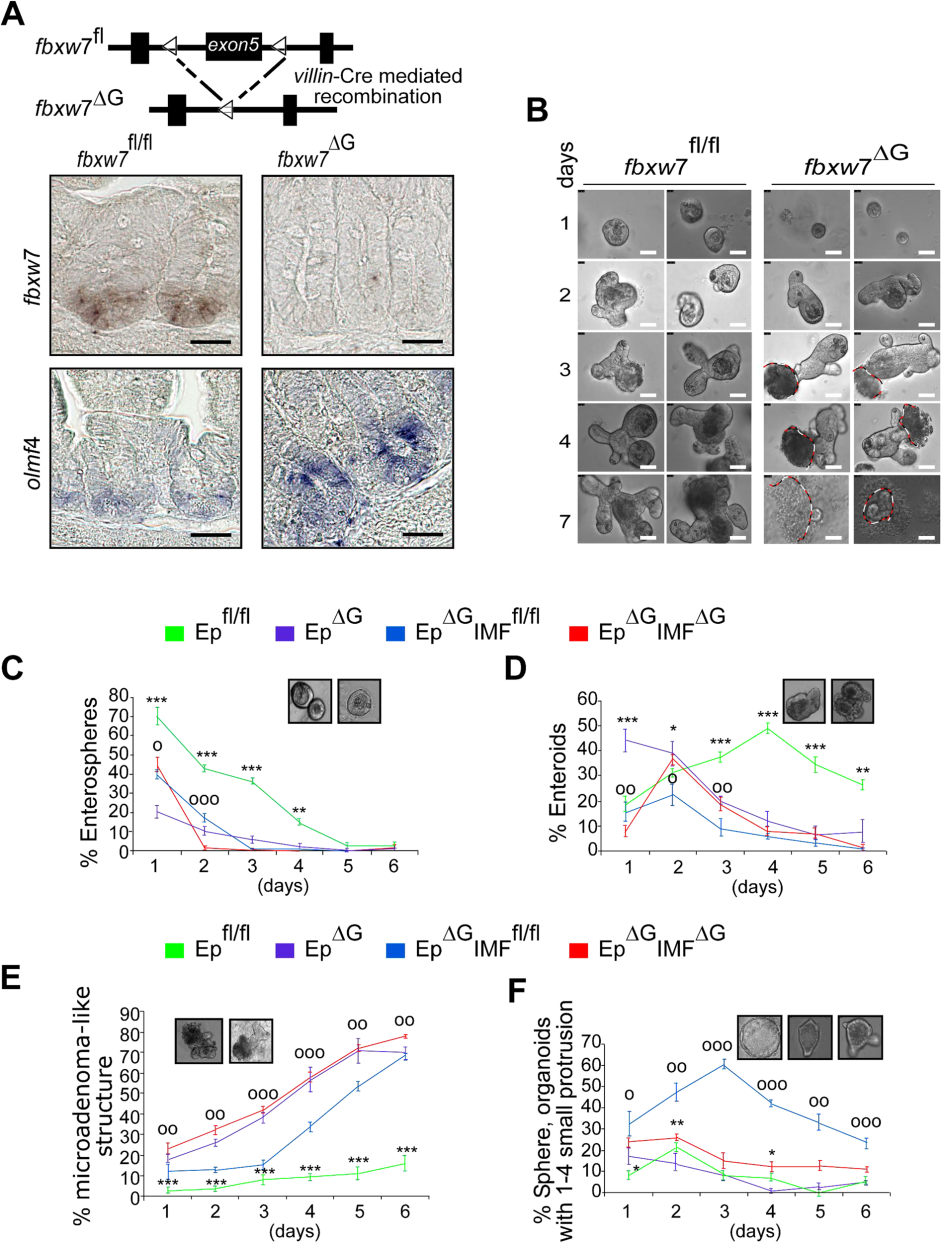
Intestinal sub-epithelial myofibroblasts (IMFs) act as a crucial extrinsic niche factor in small intestinal organoid architecture/organisation. **a** Schematic shows the *fbxw*7^fl/fl^ before and after Cre recombination to generate *fbxw*7 gut-specific inactivation (*fbxw*7^ΔG^) mice. Lower panels: ISH for *fbxw*7 and *olfm*4 mRNA on intestinal sections of 3-week *fbxw*7^fl/fl^ (left) and *fbxw*7^ΔG^ (right) mice. Scale bars, 50 μm. **b** Morphological representative images of a 7-day time course of small intestinal organoid growth from a single crypt isolated from *fbxw*7^fl/fl^ (left panels) and *fbxw*7^ΔG^ mice (right panels). Dashed lines indicate erupted epithelial cells from the *fbxw*7^ΔG^ crypts. Scale bars, 25 μm. **c**–**f** Graphs report the percentage of different morphologies found within a population of *fbxw*7^fl/fl^, *fbxw*7^ΔG^, Ep^ΔG^:IMF^fl/fl^ (*fbxw*7^ΔG^ organoids seeded on a layer of wild-type intestinal myofibroblasts) and Ep^ΔG^:IMF^ΔG^ (*fbxw*7^ΔG^ organoids seeded on a layer of *fbxw*7^ΔG^-derived myofibroblasts) organoids cultured for 1 week. Organoids were classified as enterospheres (spherical structures), enteroids (lumens and budding development with multilobulated structures), microadenoma-like structures and spheres (organoids with 1–4 small buddings). Data are from four mice per genotype with the same sex and show mean% changes over the total number of organoids in co-cultures of crypt epithelial cells and myofibroblasts (Ep:IMF), compared with a single culture of crypt epithelial cells (Ep) ± standard error of the mean (SEM) for *n* = 4 parallel wells/condition. Error bars represent SEM; (*) value Ep^fl/fl^ vs. Ep^ΔG^ and (^o^) value Ep^ΔG^:IMF^fl/fl^ vs. Ep^ΔG^:IMF^ΔG^, ***P or ^ooo^*P* ≤ 0.001; **P or ^oo^*P* ≤ 0.01; *P or ^o^*P* ≤ 0.05, as determined by Student’s *t* test

**Fig. 6:**
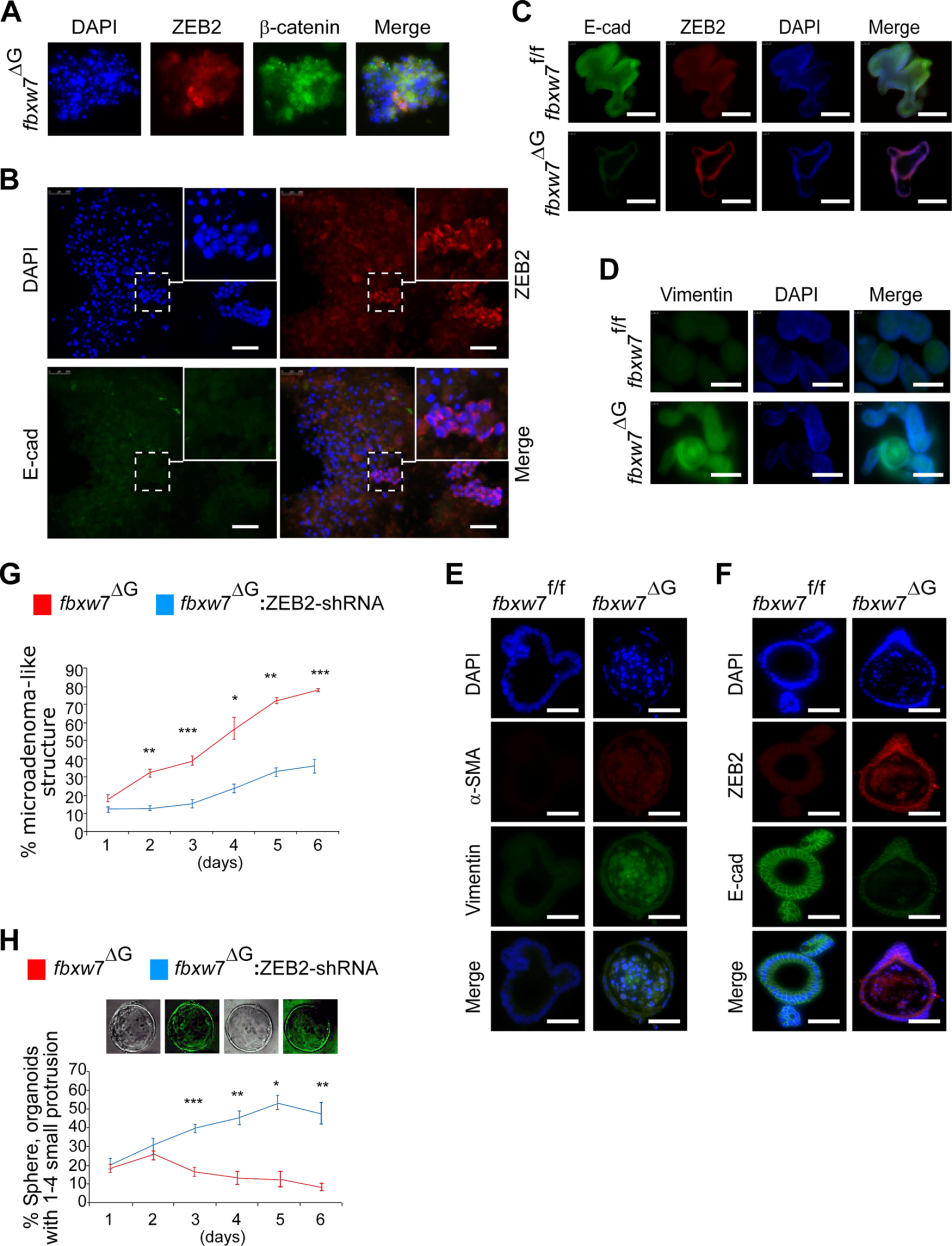
Deprivation of ZEB2 predisposes *fbxw*7-null organoids to a less malignant and more differentiated phenotype. **a** IF for ZEB2 and β-catenin detected accumulation of nuclear β-catenin with ZEB2 expressed only in a small subpopulation. Scale bars, 100 μm. **b** Increased number of erupted epithelial cells from the *fbxw*7^ΔG^ crypts after seeding in RPMI + 10% FCS medium, scale bars, 100 μm. **c**–**f** Immunofluorescence (IF) staining for ZEB2/E-cadherin (C), Vimentin (D) using paraffin sections and α-SMA/Vimentin (E) and ZEB2/E-cadherin (F) of whole-mount organoids derived from *fbxw*7^fl/fl^ and *fbxw*7^ΔG^ crypts. *fbxw*7^ΔG^ organoids lose E-cadherin expression but acquire enhanced expression of ZEB2, α-SMA and vimentin, compared with *fbxw*7^fl/fl^ controls. **g**, **h** Morphological analysis and digital quantification of *ZEB*2-shRNA:*fbxw*7^ΔG^ organoids within 6 days of growth. Murine Zeb2 knockdown of *fbxw*7^ΔG^ organoids attenuates the growth of a microadenoma-like structure and induces the formation of an enterosphere. Bars represent mean ± SD, *n* = 9; *P < 0.05, **P < 0.01, ***P < 0.001, using Student’s *t* test. Images of sphere *fbxw*7^ΔG^ organoids (**h**) shown following transduction with ZEB2-shRNA-GFP lentivirus. Experiments were performed in triplicate and repeated on two independent occasions

Previous reports showed that Fbxw7 was highly expressed in ISCs and transient-amplifying cells (TAs) in wild-type mice^5,6^. Thus, to further elucidate the physiological relevance of FBXW7/ZEB2 interaction, we used organoids (mini-gut), which are being used to model diseases including cancer. First, we found an increased expression of ISC markers, such as *Olfm*4 and *Lgr*5 in *fbxw*7^ΔG^ mice (Fig. 5a and S9, A–B). Deregulated ISCs were shown to drive the formation of tumour organoid culture^33,34^. Indeed, *fbxw7*^∆G^ organoids, but not *fbxw*7^fl/fl^-derived organoids exhibited rapid budding events in the crypt region, induction of crypt fission (Figure S9C) and microadenoma-like structures (aggregated cells from *fbxw*7^∆G^ enteroids dispersed into the culture) (Fig. 5b–f). Intriguingly, epithelial cells that had escaped from the *fbxw*7^ΔG^ microadenoma-like structures exhibited high levels of β-catenin and ZEB2 (Fig. 6a, b) and an abnormal, highly proliferative activity (Figure S9D). Furthermore, immunofluorescence (IF) staining of organoids demonstrated that Fbxw7 depletion induced an EMT (reduced E-cadherin and increased Vimentin expression) (Fig. 6c–f). We further tested the function of ZEB2 on the organoid phenotype and found that ZEB2-shRNA^K/D^ significantly decreased the number of microadenoma-like structures from *fbxw*7^ΔG^ mutant organoids, while it promoted large enterospheres versus enteroids (Fig. 6g, h). We therefore examined the effect of *ZEB*2 knockdown on the stem and secretory progenitor markers. Interestingly, *math*−1 (mouse atonal homologue-1) and *ngn*−3 (neurogenin-3) significantly induced the expression of *Olfm4* and *Lgr5* repressed in *ZEB*2-knockdown organoids (Figure S9E).

Moreover, the stromal marker, α-SMA, positively marked *fbxw*7^ΔG^ organoids but not controls (Fig. 6e). IMFs positive for α-SMA^+^ were extracted from *fbxw7*^∆G^ (IMF^∆G^) and control *fbxw7*^fl/fl^ (IMF^fl/fl^) mice, respectively (Figure S9F). The primary IMFs^∆G^ and control IMFs^fl/fl^ both showed a stellate morphology, while IMFs^∆G^ displayed a more polarised cell morphology (Figure S9F). Of note, in addition to the lower level of ZEB2 as outlined above and in Figure S8C, the expression of *interleukin*-6 (*IL*-6) was increased in isolated IMFs^∆G^ (Figure S9B), and as previously described for skin and CRCs associated with fibroblasts^35,36^. To evaluate the IMFs^∆G^ and control IMF^fl/fl^ effects on the *fbxw*7^∆G^-organoid growth, the Ep^∆G^ crypts were cocultured with IMFs^fl/fl^ or IMFs^∆G^, as feeder cells. We found that *Fbxw7*^∆G^ microadenoma-like structures were less evident during Ep^∆G^:IMF^fl/fl^ co-culture and could not continue expansion, whilst, the budding structures became limited to 1–4 small protrusions (Fig. 5e). These data suggest a role for ZEB2 signals enriched with stem-like/mesenchymal gene signatures within the *fbxw*7^ΔG^-tumour organoid microenvironment.

**Fig. 7:**
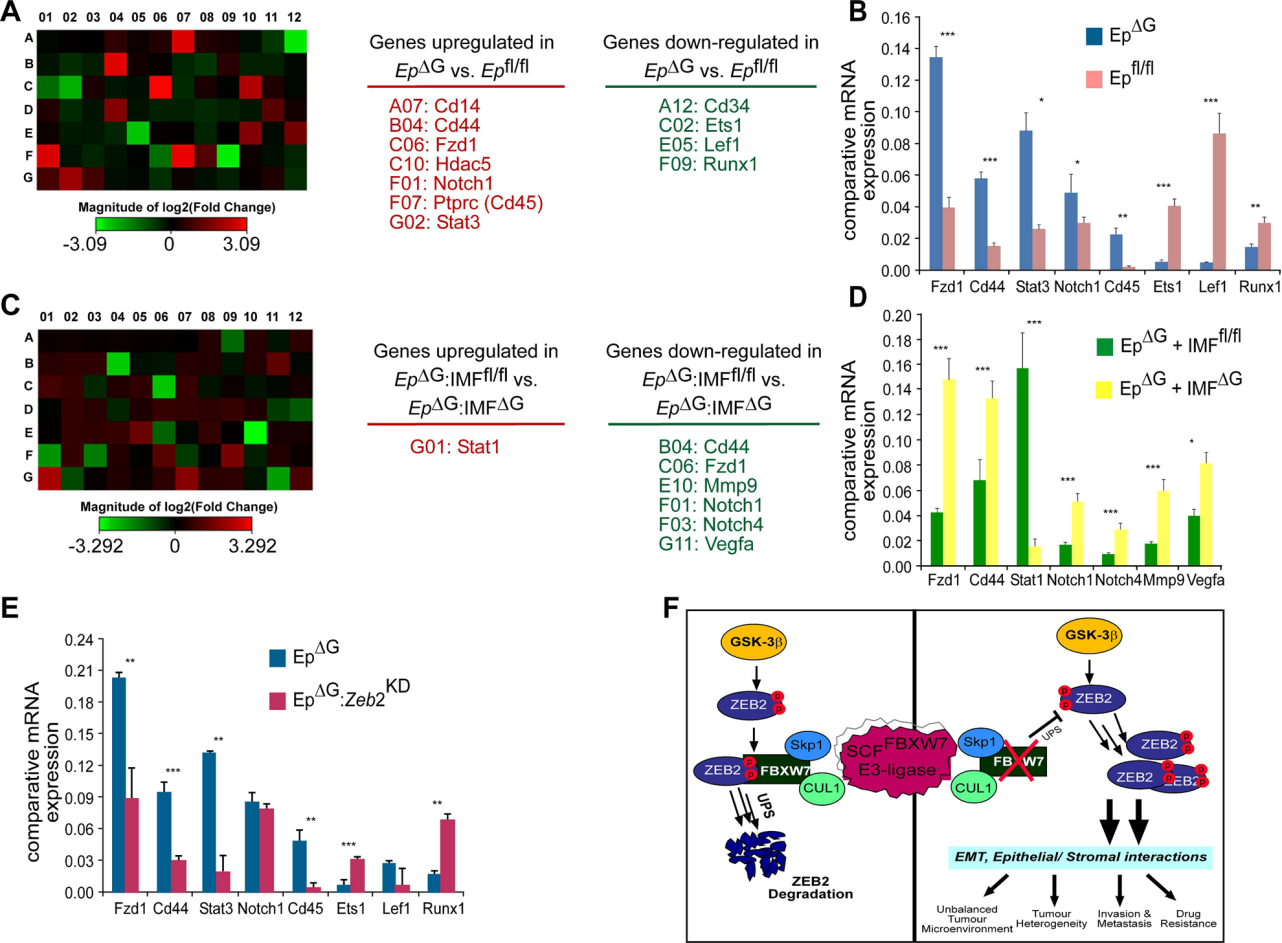
ZEB2-induced EMT and stromal factors regulate expression of a number of genes associated with stemness, invasion and anti-apoptotic response in *fbxw*7^ΔG^ organoids. **a** Heat map showing an average of 84 genes expressed from triplicate pooled samples (*n* = 25) from *fbxw*7^fl/fl^ and *fbxw*7^ΔG^ organoids (Ep^ΔG^ vs. Ep^fl/fl^) on day 1 (Table S2). Expression was determined by qRT-PCR and was first normalised to GAPDH followed by normalisation to *fbxw*7^fl/fl^ organoids. Downregulated genes (green), and upregulated genes (red). **b** qRT-PCR analysis confirming relative expression levels of a number of stem and EMT-associated genes expression in Ep^ΔG^ vs. Ep^fl/fl^ organoids. Data are mean ± SEM (*P *≤* 0.05; **P < 0.01; ***P < 0.001). Experiments were performed in triplicate for each genotype and repeated at least on three independent occasions. **c** Relative qRT-PCR transcript levels of the above-84-indicated genes from pooled samples (*n* = 15) for released Ep^ΔG^organoids from cocultured Ep^ΔG^IMF^fl/fl^ and Ep^ΔG^IMF^ΔG^ on day 3, as compiled into a heat map. Expression was normalised to GAPDH followed by normalisation to released Ep^ΔG^organoids from cocultured Ep^ΔG^IMF^fl/fl^. Downregulated genes (green), and upregulated genes (red). **d** qRT-PCR confirming relative expression levels of a number of stem and EMT-associated genes, in Ep^ΔG^IMF^ΔG^ vs. Ep^ΔG^IMF^fl/fl^ cocultured organoids. Data are mean ± SEM (**P < 0.01; ***P < 0.001). Experiments were performed in triplicate for each genotype and repeated at least on three independent occasions. **e** qRT-PCR analysis of Ep^ΔG^ vs. Ep^ΔG^:Ep^Zeb2-KD^ organoids for genes deferentially expressed in Ep^ΔG^ vs. Ep^fl/fl^ organoids. Data are mean ± SEM (*P *≤* 0.05; **P < 0.01; ***P < 0.001). Experiments were performed in triplicate for each genotype (*n* = 25) and repeated at least on two independent occasions. **f** Intestinal/colon cancer progression/metastasis could be an effect of the loss of a controlled feedback via the FBXW7/ZEB2 complex modifying EMT and epithelial–stromal interactions

Previous data showed that secreted molecules related to Wnt, TGF-β, HGF and others from fibroblasts contribute to the maintenance of CR-CSCs; we therefore performed expression profiling assay to investigate the molecular mechanisms underlying the functional interplay between fibroblasts and organoids using a cDNA array, http://www.sabiosciences.com/rt_pcr_product/HTML/PAMM-054A.html. This array allows analysis of the differential expression of 84 genes, including cytokines, signalling molecules and other regulators that are important in stemness and differentiation (Table S2A). mRNA isolated from pooled, equal numbers (25 enterospheres) of control *fbxw*7^fl/fl^ organoids and *fbxw*7^ΔG^ organoids at day 1 after seeding. Heat map analysis highlighted significant changes in associated transcripts of *fbxw*7^ΔG^ organoids versus control *fbxw*7^fl/fl^ organoids (Fig. 7a and Table S2B). Notably, several of these changes were associated with EMT/invasion (e.g. *Mmp*9, *Runx*1, *Stat*3 and *Notch*1) and Wnt signalling (e.g. *Fzd*1, *Lef*1, *Cd*44 and *Cd*45) genes (Fig. 7a and Table S2). Individual gene expression patterns were confirmed by qRT-PCR (Fig. 7b).

We then conducted a similar cDNA array, where *fbxw*7^ΔG^ organoids cocultured with IMF^fl/fl^ and IMF^ΔG^ fibroblasts, respectively, at day 1 (Ep^ΔG^IMF^fl/fl^-derived organoids vs. Ep^ΔG^IMF^∆G^-derived organoids). Remarkably, Ep^ΔG^ organoids released from cocultured Ep^ΔG^IMF^fl/fl^ showed a different gene expression pattern with Ep^ΔG^ organoids released from cocultured Ep^ΔG^IMF^∆G^ (Fig. 7c). The IMF^fl/fl^ cocultured decreased expression of some of the above-identified cytokines and Wnt/β-catenin targets, including *Mmp*9, *Fzd*1, *Wnt*3a and *Cd*44, as well as the associated transcription factors and cell-fate regulators, including *Ets*1, *Stat*1, *Stat*3, *Notch*1, *Notch*4 and *Vegfa* (Fig. 7c), whose differential expression was verified by qRT-PCR (Fig. 7d). Finally, to confirm whether ZEB2 plays a direct functional role in SC-niche activity within primary intestinal *fbxw*7^ΔG^ organoids, differential expression of the above genes in *fbxw*7^ΔG^ organoids was compared with ZEB2-shRNA^K/D^:*fbxw*7^ΔG^ organoids (Fig. 7e). Expression of a subset of genes (*Fzd*1, *Cd*44, *Stat*3 and *Cd*45) was suppressed by the *Zeb*2 knockdown, while expression of others (Est1 and Runx1) was increased or unaffected (*Notch*1 and *Lef*1; Fig. 7e). These results support the notion that ZEB2-mediated induction of EMT associated with both stromal factors secreted from cancer-like IMF^∆G^ fibroblasts and the SC-gene signature, reminiscent of the alteration of intestinal epithelial homeostasis and oncogenesis caused by Fbxw7 depletion in vivo (Fig. 7f).

## Discussion

We have identified a novel mechanism in which loss of FBXW7 influences the epithelial–stromal microenvironmental interactions, increases EMT, CR-CSC properties and metastasis.

First, we found that FBXW7 influences ZEB2 levels. Previous reports have demonstrated that ZEB2 was post transcriptionally downregulated via miRNAs^37,38^. However, our data suggest that the expression level of *ZEB2* mRNA is not altered in FBXW7-deficient cells. In contrast, GSK-3β-mediated phosphorylation of conserved sites within the homeobox-C-terminal domain of ZEB2 is not only responsible for FBXW7 binding, but also for FBXW7-mediated ubiquitination and degradation. Our biochemical analysis confirms recent data showing that other EMT-regulating transcription factors (Twist, Slug, Snail and Zeb1) are also substrates for GSK-3β^39,40^. However, further studies are needed to explore whether, like ZEB2, these proteins are substrates for and regulated by FBXW7-mediated proteasomal degradation. It is also exciting to explore the significance of GSK3β, its active (i.e. phospho-GSK3β^Tyr216^) versus inactive form (i.e. phospho-GSK3β^Ser9^) in the regulation of FBXW7-induced ZEB2 degradation and their correlation with clinical markers in the tumours (e.g. primary, advanced) and their normal counterparts.

It is widely accepted that ZEB2 was involved in cancer cell invasion, mainly through induction of EMT^18,41–43^. In addition, ZEB2 has been shown to mediate cell-fate decision in neuronal and haematopoietic stem cells^21,44^. Observations from our differential expression study also indicate the expression of ZEB2 in the ISC niche, within the intestinal epithelium, and IMFs. In addition, we showed that murine Zeb2 upregulated in crypt cells, including Paneth cells and ISCs, and dramatically downregulated within IMF in *fbxw*7-deficient mice and CRC patients with *FBXW*7 mutations. Interestingly, all the RT-PCR data suggest that ZEB2 protein is altered in a transcription-independent manner. Also, considering that the *Villin*-Cre transgene is active in intestinal epithelial cells, and with no expression in IMF cells, we reasoned that ZEB2 protein in IMF is insensitive to FBXW7-mediated degradation. Therefore, other biochemical/cellular mechanisms may change the ZEB2 protein in intestinal fibroblasts, for example, via the cytokine-mediated expression/proteolysis or translational control of protein synthesis. However, the loss of ZEB2 in stromal cells may also have paracrine effects on the epithelial cells and vice versa^45^, a mechanism that may link these two cell populations to pathological processes.

Previous studies showed that stromal factors secreted by mesenchymal/fibroblasts regulate the maintenance of stem cells, colorectal CSCs and metastatic process through a variety of signalling pathways^46–48^. Indeed, in our study, co-culture of crypts and IMF from *fbxw*7-mutant mice exerted an adverse effect on crypt development and expansion into organoids. Although the above findings have identified a role for ZEB2 in metastatic progression, it remains unclear, however, whether the resistance to chemotherapy conferred by the tumour microenvironment utilises shared or distinct molecular pathways. IMFs lacking ZEB2 may have elevated levels of the Wnt antagonist, SFRP-1^49^, and thus become incapable of supporting the normal formation of the crypt–villus compartments in the organoid.

Traditionally, our understanding of CRC is based on the analysis of aberrations within the epithelial tumour cells. Although there are no published reports that FBXW7 mutated in the CRC stroma, research shows that stromal mutations can promote tumours in genetically at-risk tissue in other systems^50,51^. The non-epithelial cell types can also be appropriately activated in response to external stimuli, such as wounding and inflammation, and inappropriately activated in cancer. For example, deletion of the murine LKB1 tumour-suppressor gene in myofibroblasts results in gastrointestinal (GI) polyposis^51^. Furthermore, TGFβ-R2 deletion in these fibroblasts leads to epithelial alterations in gastric squamous cell carcinoma^52^. Similarly, blocking stromal BMP4 signals in epithelial cells leads to adenoma-like lesions and deletion of murine Smad4 in T cells results in GI cancer^53^. Therefore, alterations in signalling from the fibroblasts may also contribute to tumour progression in CRC. Indeed, we found a significant change in ZEB2 protein expression between stromal and epithelial cell populations in *fbxw7*-knockout mice, indicating that subsequent reciprocal stromal–epithelial interactions may differentially contribute to FBXW7-deficient epithelial tumour cells. The growth of the organoid-collapsed cells reveals a novel mechanism in FBXW7-mediated ZEB2-EMT for tumorigenesis and metastasis. This may also trigger the non-epithelial-mediated rapid intestinal tumour development in double-mutant *Apc*^Min^*fbxw*7^∆G^ mice at 2–3 weeks of age^5,6^.

Consistent with recent reports on other EMT factors^54,55^, we show that FBXW7/ZEB2-regulated EMT was implicated in the early stages of metastasis and/or cancer recurrence changes by disrupting the normal balance between differentiation and drug resistance of cancer cells, which is linked to the stem-like nature of cancer cells undergoing EMT. More recent data showed that the FBXW7-ZEB1 axis is also important in cholangiocarcinoma metastasis by regulating EMT^56^. Our biochemical analysis has also confirmed an increased level of ZEB1 in FBXW7(−/−) CRC cell lines and *fbxw*7^∆G^ crypts (Figures S4C, S4D and 3A). We have also shown that the ZEB2 knockdown had partial effects in regulating E-cadherin and Vimentin [Fig. 3c (right panels) and S5E]. Therefore, we think that an individual action of these EMT-activating transcription factors may ultimately lead to a partial or steady-state level of E/M transition in cells/patients with altered FBXW7 expression. Also, as outlined above, ZEB1 is a GSK substrate for phosphorylation^40^. While recent data suggest that a partial EMT has been implicated in tumour progression and metastasis^57^, and therefore, a more in-depth investigation of EMT-TFs, such as ZEB1, Snail, Twist and Slug is required to provide full insights into the regulation of the (partial) EMT/MET process by FBXW7. Beyond this single study, an in vivo study of the role of ZEB2 in normal intestinal homeostasis and tumour initiation requires the use of multiple genetically modified mouse models, including intestinal and fibroblast ZEB2-conditional knockout and xenotransplantation into immunodeficient mouse models. Also, to identify the large scale of target genes regulated by ZEB2 in a FBXW7-dependent and FBXW7-independent manner in patients and how the epithelial–stromal alteration and interactions affect the normal homeostasis and CRC cancer initiation/progression, will require analyses of multiple primary colonospheres/organoids derived from FBXW7-deficient patients and proficient counterparts following the ZEB2 knockdown or ZEB2 knockout and/or a meaningful ZEB2 overexpression. Hence, a comprehensive genome-wide analysis can go a long way towards chromatin-IP sequencing (ChIP-Seq) and RNA-Seq assays of multiple samples. Also, further studies expanding the therapeutic potential of this newly identified pathway, by negatively instructing the EMT signalling pathways in stromal cells, could lead to important clinical implications.

## Materials and methods

### Mouse lines and human tissues

Fbxw7^fl/fl^ and fbxw7^∆G^ mouse models were described previously^5^. CRC specimens: 10 cases with and 11 cases without FBXW7 mutations were obtained on separate slides/sections as described previously^5,6^.

### In vivo metastasis/invasion assays

HCT116^FBXW7(+/+)^ and HCT116^FBXW7(−/−)^ cells with and/or without ZEB2-shRNA expression were injected into the spleen (0.5 × 10^6^ cells) or the tail vein (10^6^ cells) of five mice and tracked by bioluminescent imaging as previously described^58^.

### Tissue preparation, in situ hybridisation, immunohistochemistry and immunofluorescence assay

Murine intestines were prepared as described previously^5^. In situ hybridisation (ISH) assay was carried out as described previously^5^. Organoids were immuno-stained either as whole-mount samples or as paraffin sections. Samples for IHC were processed as outlined before^5,24^ and the following primary antibodies were used: ZEB2/SIP1 (H260; Santa-Cruz, or from Dr. Tulchinsky), E-cadherin (610181; BD), Vimentin (RV202; Santa-Cruz), α-SMA (ab5694; Abcam), Ki-67 (M7249; Dako) and Mucin2 (H300; Santa-Cruz). For IF, samples were exposed to goat anti-rabbit antibodies conjugated to Alexa Fluor594 (A11037; Invitrogen) and/or rabbit anti-mouse antibodies conjugated to Alexa Fluor488 (A11059; Invitrogen). Tetramethylrhodamine-B isothiocyanate (TRITC)-conjugated phalloidin (P1951; Sigma) was used to label actin filaments according to the manufacturer’s instruction.

### Isolation of small intestinal crypts and myofibroblasts (IMF), and in vitro crypt/IMF co-culture

Small intestinal crypts were isolated and cultured as previously described^24,33^. Crypts were released by incubation in 2 mM EDTA for 30 min at 4 °C, and further purified using a 70-µm cell strainer. The residue of the intestinal pieces was pre-treated with 1 ml of ice-cold 2.5% phenol red-free trypsin for 30 min, and incubated in 10 ml of Hanks (Sigma) containing 0.25% trypsin and 300 U ml^−1^ collagenase (Invitrogen) at 37 °C for 30 min. IMFs were eluted and cultured in DMEM with 10% FBS for 10 days to reach confluency, and then sub-cultured and used for experiments between passages 3 and 5. 300 crypts mixed with 25 μl of Matrigel (BD), plated in 48-well plates, and grown in 250 μl of advanced DMEM/F12 containing B27, N2 and 1.25 mM *N*-acetylcysteine supplements, 50 ng μl^−1^ EGF (Invitrogen), 10% Noggin and 10% R-spondin1-conditioned medium (in-house) upon solidification of the Matrigel. For crypt/IMF co-culture, crypts-Matrigel mix was seeded atop IMFs in 48-well plates with crypt-culture medium.

### Cell migration, wound healing, cytotoxicity assays and generation of 5-FU resistant cells

Cell migration and wound-healing assays were carried out as previously outlined^5^. For the cytotoxicity assay, cells were serum-starved for 18 h and then treated with 5-FU or Oxaliplatin (Tocris) for 72 h, and sulforhodamine-B colorimetric assay (Sigma, 230162) was performed as previously described^24,31^. HCT116^FBXW7(−/−)^ and HCT116^FBXW7(+/+)^ cell lines with or without *ZEB*2-shRNA were resistant to 5-FU generated by repeated exposure to increasing concentrations of 5-FU over 2–3 months^59^.

### Isolation of CD4+T cells from mouse intestine

As per the manufacturer's instructions (Miltenyi Biotec, #130-095-248), CD4 + T cells were isolated from the intestinal lamina propria (LP) of 9–10-week-old male mice, using the anti-CD4 (L3T4) MACS system^26^. Enriched CD4 + T cells were then labelled with PE-conjugated anti-CD4 (RM4–5), FITC-conjugated anti-CD45RB (16 A) and FITC-conjugated anti-CD25 (7D4). Subpopulations of CD4+T cells were then generated by two-colour sorting on FACSVantage (BD Biosciences) using the flow cytometry facility in the University of Nottingham. Both genotype populations were >97.0% pure on reanalysis.

### RRS screening

RRS screening of the mouse embryonic cDNA library in yeast cdc25–2 was carried out as previously described^9^. The RRS uses the yeast strain cdc25–2, which is deficient in Ras activity and cannot grow at 37 °C. In this study, cdc25–2 cells stably transformed with p*MET*3-GSK-3β. The activated form of GSK-3β induces phosphorylation of encoded myristoylated proteins through the pMyr-cDNA library. FBXW7-associated proteins can only rescue the growth of cdc25–2 cells if they interact with RasV12-FBXW7ΔF. Yeast colonies showing a galactose-dependent and efficient growth in the absence of methionine were isolated and further analysed.

### Proteomics assay

Two-dimensional gel electrophoresis was performed as previously described^5^. For an accurate determination of the ID and weight of the novel proteins, MALDI-MS provided by the protein chemistry facility with a Mass-Prep robotic liquid handling system, and a MALDI-TOF mass spectrometer (Waters Corporation) in the University of Nottingham was used. Peak lists entered into MASCOT-PMF (http://www.matrixscience.com/search_form_select.html) and ExPASy (http://www.expasy.org/tools/aldente/) database search engines (Table S1).

### Plasmids, transfection, cell culture and cell-cycle analysis

Human ZEB2 full-length cDNA was ligated into *Bgl*II and *Sal*I digestion sites of the pEGFP-C2 vector (Clontech). The same strategy was applied to generate eight GFP-ZEB2 deletions. Transfection of plasmids and the cell-cycle analysis was carried out as previously described^5^.

### Co-immunoprecipitation, HA ubiquitination (Ub) assay and western blotting

IP, Ub and WB assays were carried out as previously described^5,9^ using anti-Flag (F1804; Sigma), anti-ZEB2 (H260; Santa-Cruz or from Dr. Tulchinsky), anti-GFP (3E6; Invitrogen), anti-FBXW7/hCDC4 (PA1-23468; Thermo-Scientific), anti-E-cadherin (610181; BD), anti-Vimentin (RV202; Santa-Cruz), anti-α-SMA (ab5694; Abcam), anti-β-catenin (610154; BD), anti-GSK-3β (27C10; Cell-Signalling), anti-phospho-Ser/Thr (Abcam), p-c-MycT58/S62 (Cell-Signalling), HIF-1α (EP1215Y; Abcam), MCL-1 (PA5-64688; Invitrogen), P100 (EPR4686; Abcam), KLF5 (AF3758; R&D), anti-FBXW7 antibody (ab109617; Abcam) and anti-β-actin (ab6276; Abcam) antibodies.

### RT-PCR and quantitative RT-PCR assays

RNA was isolated using RNeasy Mini-Kit (QIAGEN) for CRC cells, or TRIZOL reagent (Sigma) for crypts, organoids and IMFs. Two micrograms of RNA was used to synthesise cDNA with the SuperScript-III First-Strand Synthesis System (Invitrogen) and oligo(dT) primers as per the manufacturer’s instructions. To release organoids/crypts from cocultured Ep^ΔG^IMF^fl/fl^ and Ep^ΔG^IMF^ΔG^ on day 3 for these experiments, we used a commercial cell recovery solution (Corning, 354253), which depolymerises Matrigel without enzymatic digests, to recover organoids/cells from Matrigel. We then washed released organoids/cells with cold PBS three times. Then, the organoids/fibroblasts were incubated with 3 mM EDTA in PBS for 15 min at 4 °C. To facilitate the organoid/crypt release from the fibroblasts, they were agitated by pipetting in 10% FBS/PBS and then filtered through a 70-µm strainer (Corning, 352350). The fraction containing mostly organoids/crypts, on top of the filter were collected, agitated again by pipetting with 10 ml of 10% FBS/PBS and then passed through the same filter. This passage was repeated three times. We then used an inverted microscope to choose the best fraction in terms of purity of the organoid/crypt concentration. Gene expression profiling of organoids was carried out according to the manufacturer’s instructions, http://www.sabiosciences.com/rt_pcr_product/HTML/PAMM-054A.html and using primers (Table S3), as previously described^5^.

## Supplementary information

Supplemental Figures legend and Tables

Figure S1

Figure S2

Figure S3

Figure S4

Figure S5

Figure S6

Figure S7

Figure S8

Figure S9
