# Supplementary material for: An FBXW7-ZEB2 axis links EMT and tumour microenvironment to promote colorectal cancer stem cells and chemoresistance
Source: Oncogenesis. 2019 Feb 19;8(3):13. doi: 10.1038/s41389-019-0125-3 (PMC6381143; doi:10.1038/s41389-019-0125-3)
Supplement: Supplementary file 1 — Supplemental Figures legend and Tables [file 41389_2019_125_MOESM1_ESM.docx]

**Supplemental Data**

**An FBXW7-ZEB2 axis links EMT and tumour microenvironment to promote colorectal cancer stem cells and chemoresistance**

Ningning Li ^1,2^**^≠^**, Roya Babaei-Jadidi ^1^**^≠^**, Federica Lorenzi ^1,3^**^≠^**, Bradley Spencer-Dene ^4^, Philip Clarke ^5^, Enric Domingo ^6^, Eugene Tulchinsky ^7^, Robert G. J. Vries ^8^, David Kerr ^9^, Yihang Pan ^2^, Yulong He ^2^, David O Bates ^5^, Ian Tomlinson ^6^, Hans Clevers^8^, and Abdolrahman S. Nateri ^1^**^*^**

*****Correspondence to: Abdolrahman S. Nateri, [a.nateri@nottingham.ac.uk](mailto:a.nateri@nottingham.ac.uk)

This file contains Supplementary Figures 1-9 legends, and Supplementary Tables 1, 2 & 3.

**Supplementary Figures legends**

**Figure S1. (A)** Ubiquitination assays in (+/−) FBXW7 HCT116 cells with immunoprecipitated anti-ZEB2 antibody and WB analysis using anti-Ubiquitin antibody. **(B)** ZEB2-GFP stability assays with 15µg/ml Cycloheximide (CHX) in HCT116 cells with and without *FBXW*7 deletion mutations (+/−). **C,** GSK-kinase activity mediated ZEB2 phosphorylation by using the endogenous ZEB2-immunoprecipitates when LiCl inhibits GSK-3 activity in DLD1^FBXW7-/-^ cells. **(D, E)** The GSK-3β/FBXW7 phosphodegron sequence present in ZEB2 protein is conserved across species. **(F)** HEK293T cells were transfected with the indicated ZEB2-deletion mutant constructs (D1-D5) together with FLAG-GSK-3β plasmid and were subjected to IB. Dashed boxes indicate the level of the ZEB2-mutant deletions in the absence and presence of FBXW7. **(G, H)** Histogram shows D5 wildtype and mutants bands intensity following normalization to β-actin. Data are shown as mean of three independent experiments ±SD.

**Figure S2. (A)** The co-IP assays between endogenous ZEB2 (using anti-ZEB2 antibody) and overexpressed FBXW7 (FLAG-FBXW7) in presence of GSK3 inhibitor-BIO, 1-methyl-BIO, a kinase-inactive analogue of BIO (10µM for 1 hour) and MG132 (10µM for 5 hours) was added prior to lysis to prevent degradation of ubiquitinated ZEB2, in cells deficient for *FBXW*7 (HCT116^FBXW7-/-^). **(B)** The co-IP assays between endogenous ZEB2 and FBXW7 in *FBXW*7 knockout cells versus *FBXW*7 wild-type cells using anti-ZEB2 antibody. **(C)** The co-IP assays between endogenous ZEB2 and FBXW7 in *ZEB*2-knockdown and wild-type cells.

**Figure S3. (A)** The HCT116 cells expressing HA-Ubiquitin, FLAG-FBXW7 and GFP-ZEB2 mutants, were lysed. An immunoprecipitation by anti-HA antibody (HA-ubiquitin) followed by immunoblotting with anti-Ubiquitin and anti-GFP (ZEB2 mutants). The ubiquitinated ZEB2-D8 was detected by GFP western blot. **(B)** Schematic of a full-length ZEB2 lacking the aa705-870 (ZEB2-ΔD8). **(C)** CHX chases assay indicates that the ΔD8 mutant stabilises the ZEB2 protein, and this further confirmed that aa705-870 residues contribute to de-stabilisation of ZEB2 protein. **(D)** The ZEB2-ΔD8 overexpression had no effects on E-cadherin and Vimentin protein levels in HCT116 cells.

**Figure S4. (A)** Protein extracts from crypts of *fbxw*7^fl/fl^ and *fbxw*7^ΔG^ mice and CRC cell lines with and without FBXW7 deletion were analyzed by Western blotting. **(B)** FBXW7, ZEB2, E-cadherin, Vimentin and GSK-3β expression profile in HCT116 cell lines expressing or lacking Fbxw7. HCT116 ^FBXW7(-/-)^ cells acquired EMT markers. β-actin was blotted for loading control. **(C)** Verification of ZEB2 antibody. FBXW7-deficient HCT116 cells show marked ZEB2 increase versus parental HCT116 ^FBXW7(+/+)^ cells (left vs. middle panels). The recombinant vector pGFP-ZEB2 was forced expressed in HCT116 cells and served as a positive control (right panels) for indirect IF staining for ZEB2. The exogenous ZEB2 shows its epitope tag colour, green, and is also detected via a red IF dye conjugated to a secondary antibody that recognizes the ZEB2 antibody. Scale bars, 100μm. **(D-F)** RT-PCR and qRT-PCR analysis of *FBXW*7, *ZEB*1, *ZEB*2 and miR200 expression in (+/−) FBXW7 CRC cells, epithelial cells and IMFs isolated from 3-wk-old *fbxw*7^fl/fl^ and *fbxw*7^ΔG^ mice. Data are mean ± SEM. Experiments were performed in triplicate for each genotype and repeated at least on three independent occasions.

**Figure S5. (A)** IHC for ZEB2 on representative images of matched adjacent normal mucosa of CRC tissues showed in Figure 3B, bottom images. Red arrowheads show Ep and green arrowheads show stromal-cells with different ZEB2 protein levels. Scale bars; 50μm. **(B)** Number of LP CD4+ T cells population derived from *fbxw*7^fl/fl^ vs. *fbxw*7^ΔG^ mice isolated from MACS-purified CD4+ T cells by FACS sorting. **(C-D)** Western blot analysis of *fbxw*7^fl/fl^ vs. *fbxw*7^ΔG^ derived crypts, IMF and (+/−) FBXW7 HCT116 cells proteins using antibodies against, Snail1, ZEB1, and the loading control β-actin.

**Figure S6. (A-D)** Comparison of ZEB2 (A), the filamentous actin cytoskeleton, stained with tetramethylrhodamine B isothiocyanate conjugated Phalloidin (TRITC-Phalloidin) (B), epithelial marker E-cadherin (C) and mesenchymal marker Vimentin (D) in shRNA knockdown of ZEB2 (ZEB2-shRNA) and scrambled (sc) in HCT116 cells with and without FBXW7 deletion. Scale bars; 25μm. Experiments were performed in triplicate. **(E)** ZEB2, E-cadherin and Vimentin expression in HCT116^FBXW7(−/−)^ cells treated with sc or si-ZEB2 by WB analysis. **(F)** RT-PCR analysis of *ZEB*2 and *FBXW*7 mRNA expression in HCT116 sc-shRNA:FBXW7(+/+), sc-shRNA:FBXW7(-/-), ZEB2-shRNA:FBXW7(+/+) and ZEB2-shRNA:FBXW7(-/-) cell lines. *HPRT* was detected as PCR control. **G,** ZEB2, E-cadherin and Vimentin proteins measured at low dose (0.25μM) and high (2.5μM) Oxaliplatin (OX) treatment in HCT116^FBXW7(+/+)^ and HCT116^FBXW7(−/−)^ cells by WB analysis.

**Figure S7.** **(A)** HCT116 cells expressing (left) or lacking FBXW7 (right) were subjected to *in vitro* transwell migration assay for 24 h after starvation overnight. Overexpression of ZEB2 results in increased migration of parental HCT116 cells; suppression of ZEB2 attenuates the migration of HCT116^FBXW7(-/-)^ cells. Graphs show mean ± SEM of assay data, representative of triplicate experiments performed for at least three times (**P* < 0.05, ***P* < 0.01, ****P* < 0.001). **(B-C)** Loss of FBXW7 induces EMT resulting in increased rate of *in vitro* wound closure and cell migration. DLD-1^FBXW7(+/+)^ and DLD-1^FBXW7(-/-)^ cells (left) and HCT116^FBXW7(+/+)^ and HCT116^FBXW7(-/-)^ cells (right) were analyzed for migration by a wound-healing assay. Cells were plated and disrupted with a 200-μl tip. 24 h after disruption, the cell layer was photographed as described. *In vitro* wound closure quantification, presented as a percentage and analyzed by subtracting the distance measured between the edges of the wound at hour 24 from that at hour 0 (B). **(D)** Representative images of immunodeficient mice following intra-splenic (left) or intravenous (right) injection of sc-shRNA:FBXW7(-/-) and ZEB2-shRNA:FBXW7(-/-) cell lines. Images were taken by Xenogen IVIS Imaging System immediately after cell administration. **(E)** Representative image was taken before termination of mice retained with metastases tumours following injections. **(F)** Lungs and liver of all mice were isolated and fixed for further analyses. **(G)** Representative images of paraffin-embedded metastasis tumours for validation/confirmation that KRT5 marker discriminates human CRC cells in murine tissues. Scale bars, 100μm.

**Figure S8.** (**A)** Top; Colony forming ability of 5-FU resistant HCT116 cell lines with and without FBXW7 deletion expressing ZEB2-shRNA and/or sc-shRNA. A significant decrease in sensitivity to 5-FU, in each drug-resistant ZEB2-shRNA cell lines relative to the sc-shRNA parental lines, when treated with a range of concentrations of 5-FU. Colonies were stained with crystal violet at day 10 of growth. Bottom; Histogram shows colony formation efficiency of sc-shRNA:FBXW7(-/-) and shRNA:FBXW7(+/+) or ZEB2-shRNA:FBXW7(+/+) and ZEB2-shRNA:FBXW7(-/-) and/or vice versa. Experiments were performed in triplicate for each cell line on two independent occasions. Data are shown as mean ± SD, n=3. P values were calculated using Student’s *t*-test (**P* < 0.05, ***P* < 0.01, ****P* < 0.001). **(B)** Synchronised/serum starved CRC cells with and without feeder human embryonic normal fibroblasts (NFs) and/or cancer-associated fibroblasts (CAFs), were treated with increasing concentrations of 5-FU; for 72h. IC50 and cell viability were determined using sulforhodamine B/Trizma base protein assay (SRB) by measuring absorbance at 540nm from six replicates in 3 independent experiments. As outlined above, *P* values were estimated using the AIC approach of Prism software. **(C)** IF of ZEB2, on primary IMFs derived from *fbxw*7^∆G^ versus *fbxw*7^fl/fl^ and grown on glass coverslips. Scale bars, 100μm.

**Figure S9. (A)** ISH for *olfm*4 on representative intestinal sections from 3-wk-old *fbxw*7^fl/fl^ (top panels) and *fbxw*7^ΔG^ (bottom panels) mice. Experiments were performed using sense and anti-sense probes and on at least two independent occasions. **(B)** qRT-PCR analysis of *fbxw*7, *olfm*4, *Lgr*5, *math1* and *IL-6* mRNA expression in epithelial and IMF from isolated crypts of 3-wk-old *fbxw*7^fl/fl^ and *fbxw*7^ΔG^ mice. Experiments were performed on at least three independent occasions. **C,** Ki-67 IHC of whole organoids derived from *fbxw*7^∆G^ or *fbxw*7^fl/fl^ mice. Boxed areas are enlarged shown in the bottom panels. Figures are representative of duplicate experiments performed on three separate occasions. Bars, 25μm. **(D)** CFSE staining transition from day 1 to day 3 of erupted culturing epithelial cells from the *fbxw*7^ΔG^ organoids. **(E)** qRT-PCR analysis of *zeb2*, *olfm*4, *Lgr*5, *math1* and *ngn3* mRNA expression in *fbxw*7^ΔG^ and *fbxw*7^ΔG^:*Zeb*2-knockdown organoids. Experiments were performed in triplicates and on two independent occasions. **(F)** IF of α-smooth muscle actin (α-SMA), on primary IMFs derived from *fbxw*7^∆G^ versus *fbxw*7^fl/fl^ grown on glass coverslips. Both types of IMFs are α-SMA^+ve^ but show distinct morphology. The boxed line indicates magnified cells. Scale bars, 100μm.

**Supplementary Tables**

**Table S1: Identification of differentially expressed spots in *fbxw*7^fl/fl^ and *fbxw*7^ΔG^ isolated intestinal mouse crypts using MALDI-TOF mass spectrometry and significance threshold p<0.05.** Fbxw7 associated proteins (FAPs) that were identified by both RRS and 2D- MALDI-MS assays and had no prior reports of a functional link with FBXW7 highlighted in blue.

| **Spot**  **protein (ID)** | **Accession no. (Swiss-Prot)** | **Mass (Da)** | **Sequence**  **Coverage (%)** | **MASCOT**  **score** | **Ref.** |
| --- | --- | --- | --- | --- | --- |
| Deptor | Q570Y9 | 46,120 | 19 | 89 | Mol Cell 44, 290-303 (2011). |
| Integrin a-3 | Q62470 | 113,428 | 13 | 58 | Curr Opin Cell Biol. 13, 541-545 (2001). |
| Rad51 | Q08297 | 36,971 | 21 | 67 | Semin Cell Dev Biol. 22, 898-905 (2011). |
| Elk1 | P41969 | 45,271 | 34 | 88 | Biochim Biophys Acta. 1829, 1026-1033 (2013). |
| Fabp6 | P51162 | 14,486 | 28 | 36 | Cancer Res. 12, 3352-3363 (2017). |
| Klf9 | O35739 | 27,170 | 48 | 133 | Physiological reviews 90, 1337-1381 (2010). |
| DEK | Q7TNV0 | 43,159 | 35 | 66 | Cell cycle 12, 51-66 (2013). |
| Zswim2 | Q9D9X6 | 71,793 | 16 | 101 | Science 309, 1559-1563 (2005). |
| Prdx6 | O08709 | 24,871 | 29 | 93 | Methods Enzymol. 527, 145-167 (2013). |
| Zeb2 | Q9R0G7 | 136,615 | 39 | 79 | Cell Mol Life Sci 66, 773-787 (2009). |
| Cdc16 | Q8R349 | 71,460 | 23 | 56 | J Cell Biochem. 103, 1327-1343 (2008). |
| Fgf1 | P61148 | 17,418 | 15 | 87 | J Cell Biochem, 103(5), 1327-43 (2008). |
| Elk3 | P41971 | 44,414 | 19 | 71 | Oncogene, 19(55), 6524-32 (2000). |
| Prdm6 | Q3UZD5 | 64,503 | 43 | 52 | Mol Cell Biol. 26, 2626-2636 (2006). |
| Foxm1 | O08696 | 83,694 | 29 | 96 | Front Oncol. 3, 30 (2013). |
| Hp1bp3 | Q3TEA8 | 60,867 | 23 | 69 | Nucleic Acids Res, 43, 2074-90 (2015). |
| Hes6 | Q9JHE6 | 24,454 | 33 | 84 | Mech Dev. 98, 133-137 (2000). |
| Ppp2r5a | Q6PD03 | 56,347 | 18 | 69 | J Mol Biol. 336, 971-986 (2004). |
| Timp4 | Q9JHB3 | 25,774 | 12 | 54 | FEBS letters 401, 213-217 (1997). |
| Tada2a | Q8CHV6 | 51,339 | 16 | 56 | Proc Natl Acad Sci U S A, 103(7), 2057-62. (2006). |
| Nomo1 | Q6GQT9 | 133,420 | 26 | 68 | RNA biology 9, 1002-1010 (2012). |
| Csnk1a1 | Q8BK63 | 38,915 | 37 | 68 | DNA Seq 8, 55-57 (1997). |
| Znrf3 | Q5SSZ7 | 98,967 | 25 | 74 | Nature 485, 195-200 (2012). |
| Nanog | Q80Z64 | 34,240 | 29 | 56 | Stem Cells, 30, 2076-2087, (2012). |
| Rnf146 | Q9CZW6 | 38,934 | 18 | 90 | Nat Cell Biol, 13, 623-929 (2011). |
| Glis1 | Q8K1M4 | 84,172 | 24 | 76 | Nature 474, 225-229 (2011). |
| Nek9 | Q8K1R7 | 107,143 | 16 | 82 | Journal of cell science 125, 4423-4433 (2012). |

**Table S2, A)** Gene expression profiling of organoids; Ep^ΔG^ vs. Ep^fl/fl^

**Table S2, B)** Gene expression profiling of co-cultured organoids; Ep^ΔG^IMF^ΔG^ vs. Ep^ΔG^IMF^fl/fl^

**Table S3. Sequences of PCR primers are as follows:**

5′-AATGCACAGAGTGTGGCAAGGC (human ZEB2 forward)

5′-CTGCTGATGTGCGAACTGTAGG (human ZEB2 reverse)

5′-GCAGTGAGCATCGAAGAGTACC (mouse Zeb2 forward)

5′-GGCAAAAGCATCTGGAGTTCCAG (mouse Zeb2 reverse)

5′-GGCATACACCTACTCAACTACGG (human ZEB1 forward)

5′-TGGGCGGTGTAGAATCAGAGTC (human ZEB1 reverse)

5′-ATTCAGCTACTGTGAGCCCTGC (mouse Zeb1 forward)

5′-CATTCTGGTCCTCCACAGTGGA (mouse Zeb1 reverse)

5′-AGCTGTCCAAATATGAGACCCTACA (Math1 forward)

5′-GACATTGGGAGTCTGCAGCAA (Math1 reverse)

5′-GAGACTTCATCTCCTTGCTTCCTAAA (Fbxw7 forward)

5′-CGCTTGCAGCAGGTCTTTG (Fbxw7 reverse)

5′-CGGTCGATGCAACGAGTGATGAGG (Cre forward)

5′-CCAGAGACGGAAATCCATCGCTCG (Cre reverse)

5′-CAGTGGAGTGAAGTACAACTC (Floxed Fbxw7 forward) (WT, 287nt; floxed, 394nt)

5′-GCATATTCTAGAGGAGGGTAT (Floxed Fbxw7 reverse) (WT, 287nt; floxed, 394nt)

5′-CGGAGGAAGCGCTACAGAAT (Lgr5 forward)

5′-CTGGGTGGCACGTAGCTGAT (Lgr5 reverse)

5′-CCCCCTTTCTTTTTCCAGTT (Cd44 forward)

5′- ACTTTCTGCCCCTCTCCACT (Cd44 reverse)

5′- AATCTCTTCTAGAGACTGGGAAGGAG (Mmp9 forward)

5′- AGCTGATTGACTAAAGTAGCTGGA (Mmp9 reverse)

5′- AGGTGACAGCATTGCTTCTG (forward β-actin)

5′- AGGGAGACCAAAGCCTTCAT (reverse β-actin)

5′- GTCCGAAGTGTTACCCTGGA (forward Muc2)

5′- CCAGGAGTGGAGAAGGTCAG (reverse Muc2)

5′- TCAATGCCGTGGATGACCTA (forward Notch1)

5′- CCTTGTTGGCTCCGTTCTTC (reverse Notch1)

5′- TTCCCAAAAGGAGCCTCTGC (forward Notch4)

5′- TCTACACAACACCCGGCACA (reverse Notch4)

5′- TTATGCCATCCCTTGCCCTCACC (forward Fzd1)

5′- GGTAAGCCTCGTGTAGAACTTCC (reverse Fzd1)

5′- GATCCACTGCTGGGCTTCA (forward Cd45)

5′- GAACATGCTGCCAATGGTTCT (reverse Cd45)

5′- GACCCGCCAACAAATTAAGA (forward Stat3)

5′- TCGTGGTAAACTGGACACCA (reverse Stat3)

5′- AAGCGAACTGGATACATCA (forward Stat1)

5′- CCGGGACATCTCATCAAAC (reverse Stat1)

5′- AGTGGACAGAAACCCATGTT (forward Ets-1)

5′- CAAAGTCTGGGGCCAGCT (reverse Ets-1)

5′- CCACACGGACAGTGACCTA (forward Lef1)

5′- TGGGCTCCTGCTCCTTTCT (reverse Lef1)

5′-CCTCCTTGAACCACTCCACT (forward Runx1)

5′-CTGGATCTGCCTGGCATC (reverse Runx1)

5′-CTGTGCAGGCTGCTGTAACG (forward Vegf-A)

5′-GTTCCCGAAACCCTGAGGAG (reverse Vegf-A)

5′-CTCTGCAAGAGACTTCCATCCAGT-3′(forward IL-6)

5′-GAAGTAGGGAAGGCCGTGG-3′(reverse IL-6)
