# Supplementary figures and images for: An FBXW7-ZEB2 axis links EMT and tumour microenvironment to promote colorectal cancer stem cells and chemoresistance
Source: Oncogenesis. 2019 Feb 19;8(3):13. doi: 10.1038/s41389-019-0125-3 (PMC6381143; doi:10.1038/s41389-019-0125-3)

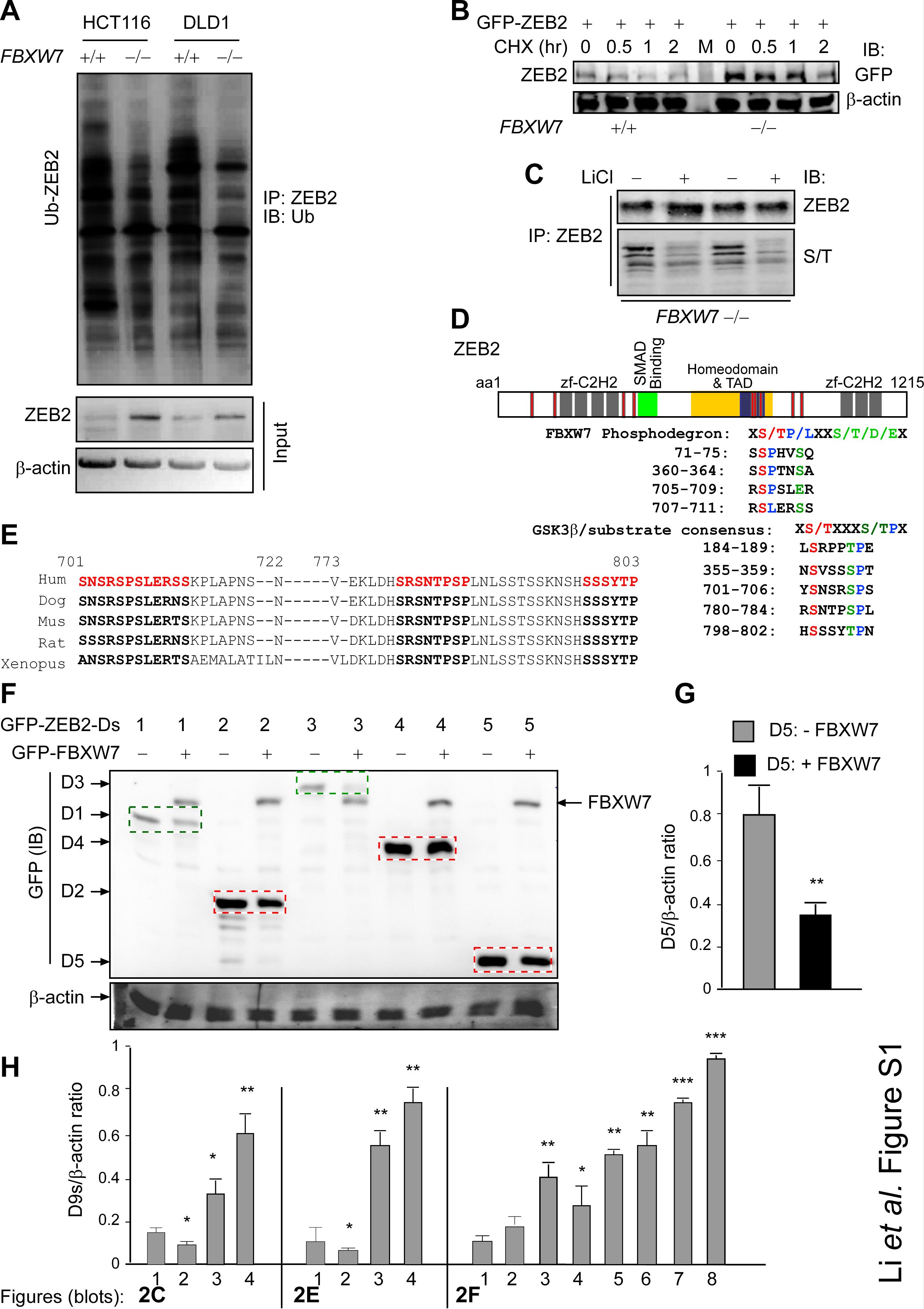

Supplement: Supplementary file 2 — Figure S1 [file 41389_2019_125_MOESM2_ESM.jpg]

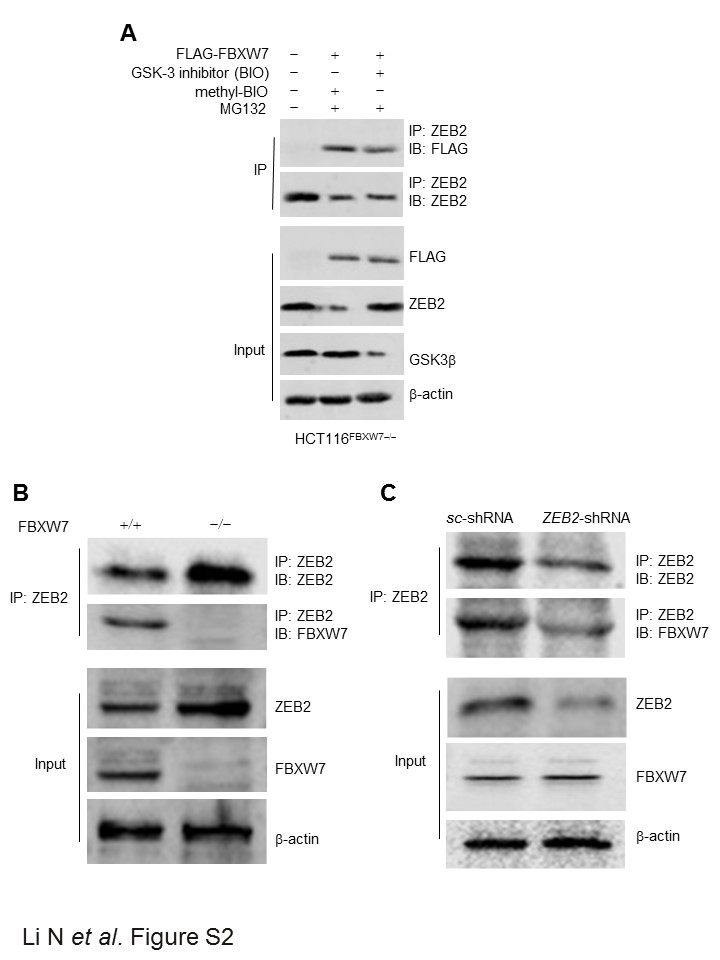

Supplement: Supplementary file 3 — Figure S2 [file 41389_2019_125_MOESM3_ESM.jpg]

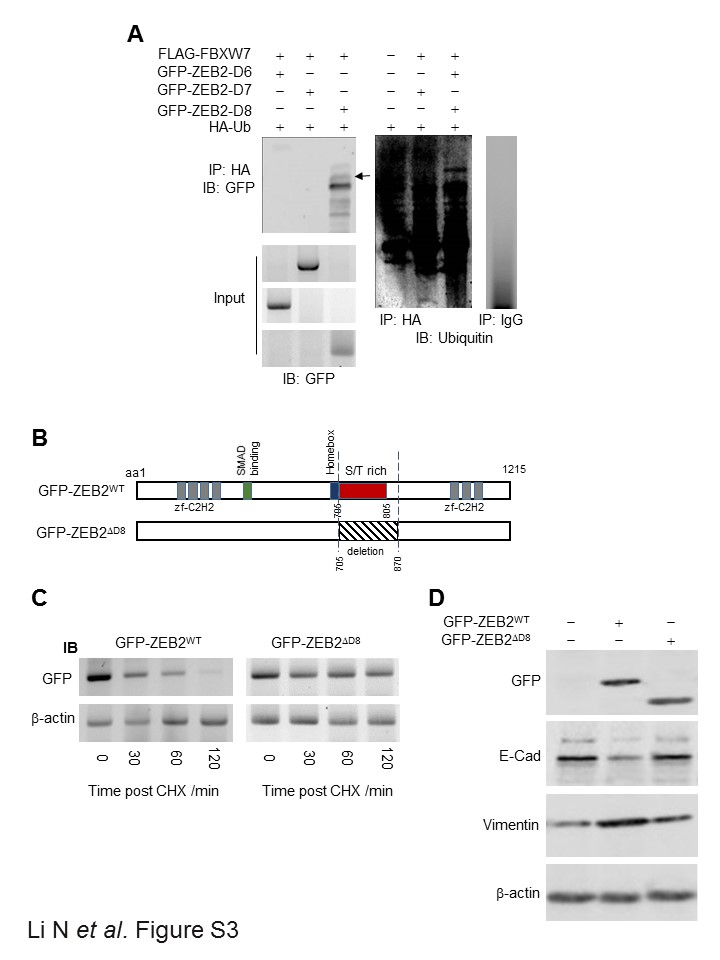

Supplement: Supplementary file 4 — Figure S3 [file 41389_2019_125_MOESM4_ESM.jpg]

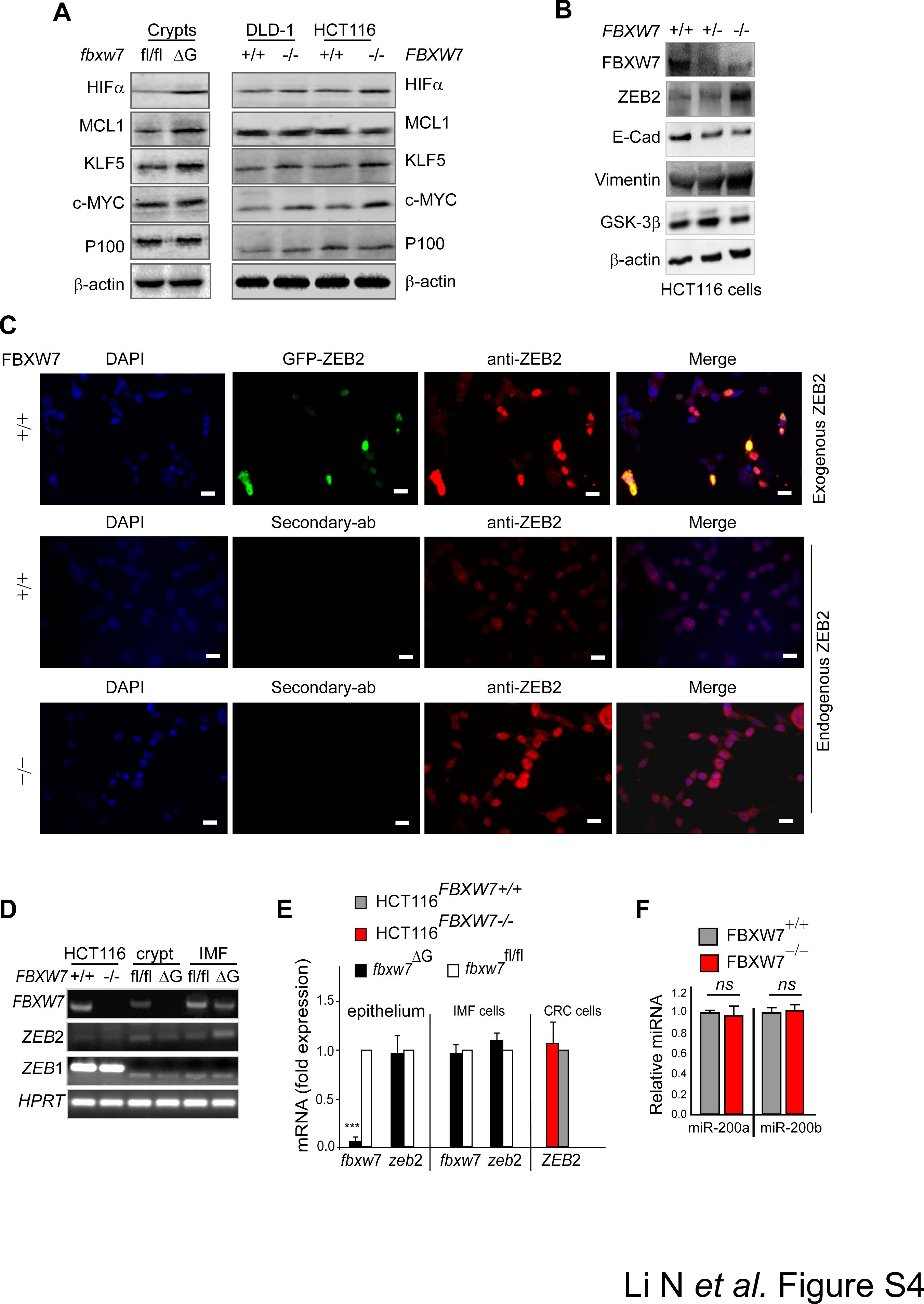

Supplement: Supplementary file 5 — Figure S4 [file 41389_2019_125_MOESM5_ESM.jpg]

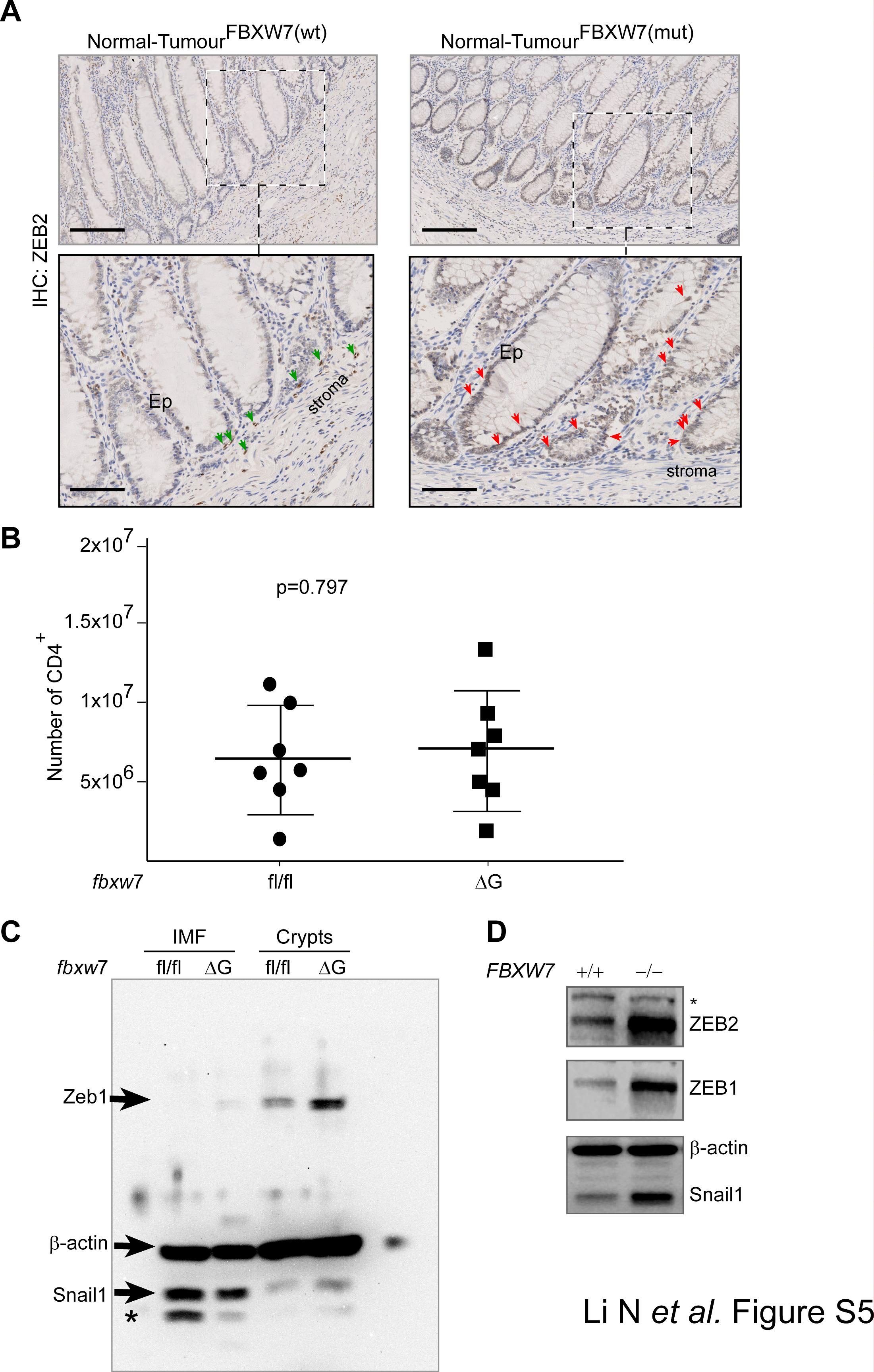

Supplement: Supplementary file 6 — Figure S5 [file 41389_2019_125_MOESM6_ESM.jpg]

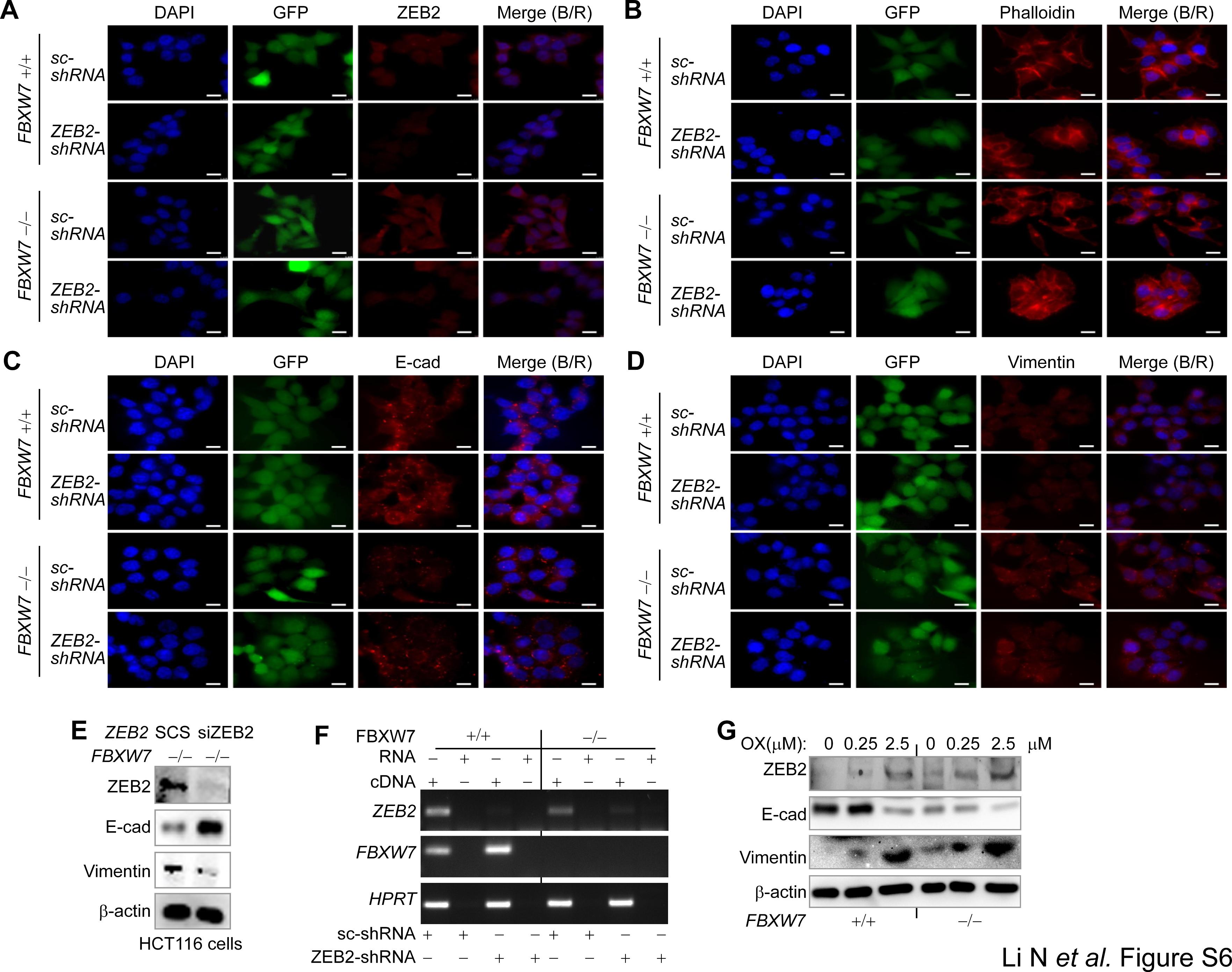

Supplement: Supplementary file 7 — Figure S6 [file 41389_2019_125_MOESM7_ESM.jpg]

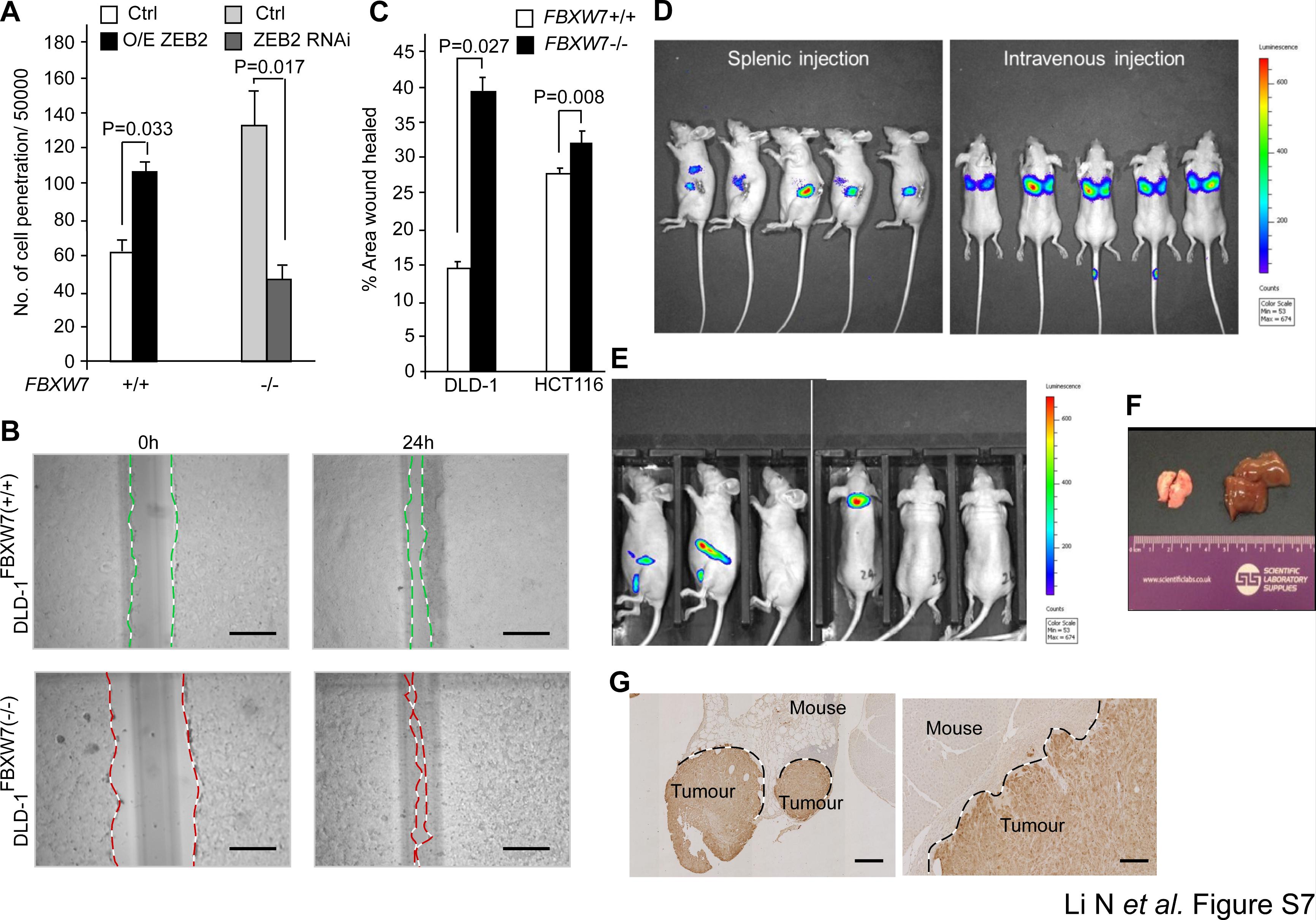

Supplement: Supplementary file 8 — Figure S7 [file 41389_2019_125_MOESM8_ESM.jpg]

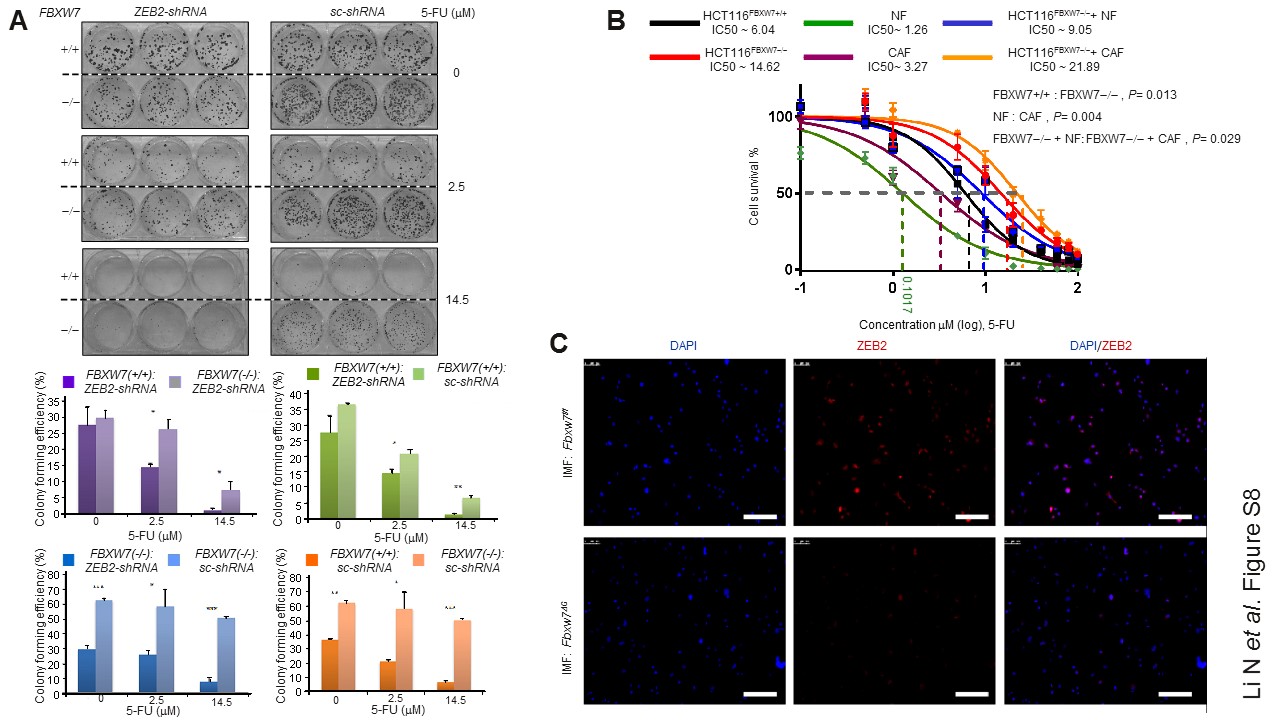

Supplement: Supplementary file 9 — Figure S8 [file 41389_2019_125_MOESM9_ESM.jpg]

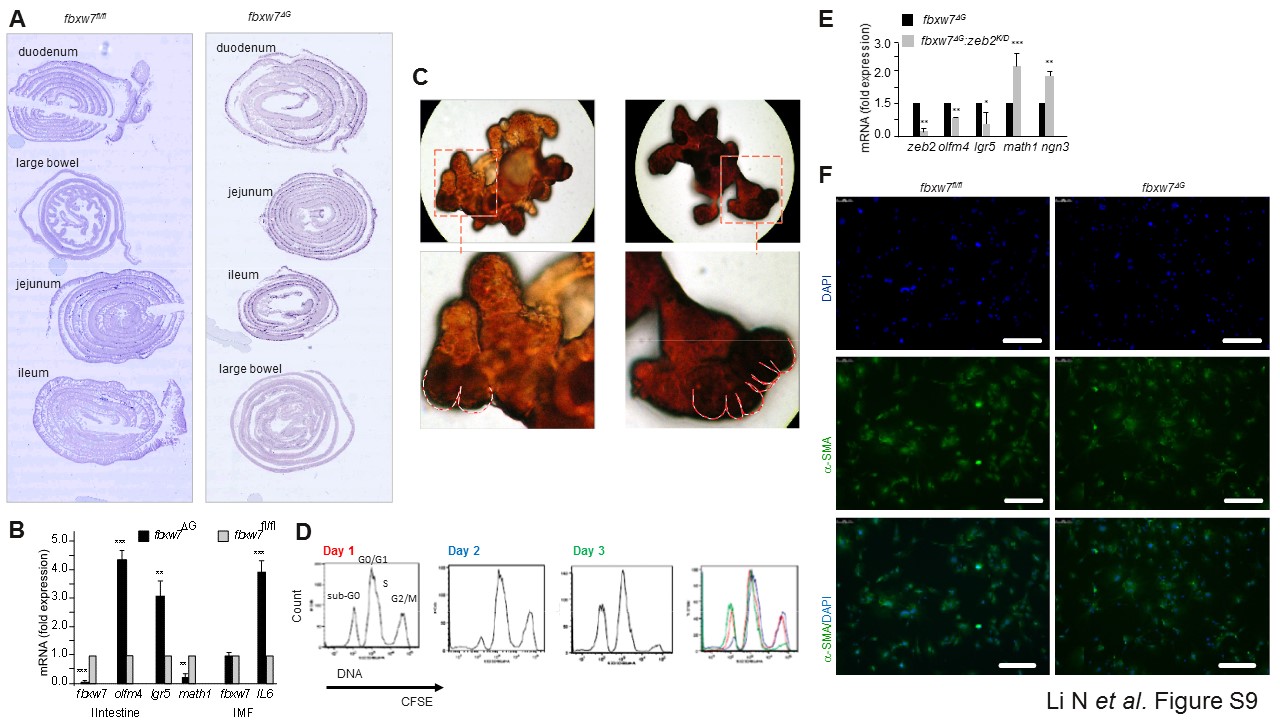

Supplement: Supplementary file 10 — Figure S9 [file 41389_2019_125_MOESM10_ESM.jpg]
